# Maternal exposure to fine particulate matter and pregnancy outcomes in women undergoing *in vitro* fertilization: A multicenter retrospective study

**DOI:** 10.1016/j.eehl.2025.100192

**Published:** 2025-10-15

**Authors:** Miaoxin Chen, Ying Fang, Yunxiu Li, Guimin Hao, Xueqing Wu, Yan Sun, Jichun Tan, Yue Niu, Xinyi Du, Yonggang Li, Zhuoye Luo, Fen Hu, Yuehong Li, Shanshan Wu, Yingying Yang, Orhan Bukulmez, Yeung William Shu-Biu, Robert J. Norman, Haidong Kan, Xiaoming Teng

**Affiliations:** a Center for Assisted Reproduction, Shanghai Key Laboratory of Maternal Fetal Medicine, Shanghai Institute of Maternal-Fetal Medicine and Gynecologic Oncology, Shanghai First Maternity and Infant Hospital, School of Medicine, Tongji University, Shanghai 200092, China; b School of Public Health, Key Lab of Public Health Safety of the Ministry of Education, NHC Key Laboratory of Health Technology Assessment, Fudan University, Shanghai 200032, China; c Department of Reproductive Medicine, The First People's Hospital of Yunnan Province, NHC Key Laboratory of Preconception Health Birth in Western China, Kunming 650032, China; d Department of Reproductive Medicine, The Second Hospital of Hebei Medical University, Shijiazhuang 051161, China; e Center of Reproductive Medicine, Children's Hospital of Shanxi and Women Health Center of Shanxi, Taiyuan 030013, China; f Center of Reproductive Medicine, Fujian Maternity and Child Health Hospital, College of Clinical Medicine for Obstetrics & Gynecology and Pediatrics, Fujian Medical University, Fuzhou 350001, China; g Center for Reproductive Medicine, Department of Obstetrics and Gynaecology, Shengjing Hospital of China Medical University, Shenyang 110022, China; h Division of Reproductive Endocrinology and Infertility, Department of Obstetrics and Gynecology, The University of Texas Southwestern Medical Center, TX 75390, USA; i Shenzhen Key Laboratory of Fertility Regulation, Center of Assisted Reproduction and Embryology, The University of Hong Kong–Shenzhen Hospital, Shenzhen 518053, China; j Robinson Research Institute, Adelaide Medical School, The University of Adelaide, SA 5005, Australia; k Children's Hospital of Fudan University, National Center for Children's Health, Shanghai 201102, China

**Keywords:** Fine particulate matter, *In vitro* fertilization, Live birth, Pregnancy outcomes

## Abstract

Few large-scale studies have systematically examined the effects of maternal exposure to fine particulate matter (PM_2.5_) on live birth in women undergoing *in vitro* fertilization (IVF). This study aimed to investigate the associations between ambient PM_2.5_ exposure and live birth in women treated with IVF, and determine critical periods, key failure events, and vulnerable populations affected by such exposure. We included 58,637 patients from six reproductive centers in China between 2016 and 2021. We defined six exposure windows and adopted logistic regression with random-effect models to investigate the associations between PM_2.5_ exposure and live birth. We further categorized live birth failure as implantation failure, biochemical pregnancy loss, and miscarriage, to determine at which stage PM_2.5_ exposure caused the live birth failure. Subgroup analyses were conducted by female age, ovarian response, embryo quality, and transplantation protocol. For each 10 ​μg/m^3^ increase in PM_2.5_ concentration during follicle growth phase, preantral-antral follicle phase, and antral-mature follicle phase, the odds ratios for live birth were 0.966 [95% confidence interval (CI): 0.938, 0.995], 0.967 (0.939, 0.996), and 0.978 (0.958, 0.998), respectively. PM_2.5_ during these stages was significantly associated only with an increased likelihood of implantation failure, highlighting adverse impact of ambient PM_2.5_ on early pregnancy outcome. In addition, we observed relatively stronger associations in women with poor ovarian response, compromised embryo quality, and those undergoing fresh or double embryo transfers. This large-scale population-based study demonstrated the detrimental effects of high PM_2.5_ exposure for IVF women, shedding light on clinical and public health practices.

## Highlights

• Maternal high PM_2.5_ exposure in follicular growth stages were significantly associated with decreased live birth.
• The PM_2.5_-associated live birth failures primarily arise in the early pregnancy stages.
• Women with poor ovarian response, diminished embryo quality, undergoing fresh or double embryo transfers may be more sensitive to PM_2.5_ exposure.

## Graphical abstract

**Figure:**
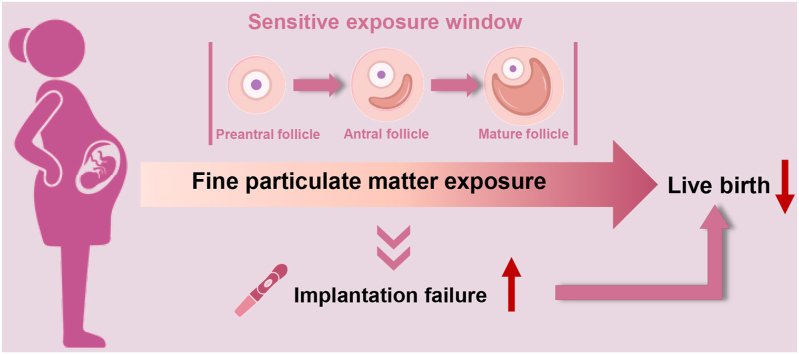

## 1 Introduction

*In vitro* fertilization (IVF), initially developed for couples with tubal infertility issues, has become a well-established medical procedure for treating prolonged unresolved infertility [1]. In 2019, it was estimated that over 2.5 million IVF cycles were performed worldwide annually [2]. Given that the success of IVF treatment is not guaranteed, identifying its modifiable risk factors is crucial for clinical practice and public health. Particulate matter with a diameter ≤ 2.5 ​μm (PM_2.5_) is a widespread air pollutant and has been linked to extensive health impairments, including adverse pregnancy and birth outcomes [[3], [4], [5]]. The underlying biological mechanisms, such as increased oxidative stress, inflammation, and epigenetic modification in oocytes or placentas, have been documented [[6], [7], [8], [9]]. Compared to the non-IVF conception group, women who conceived through IVF are generally considered to be more susceptible to environmental stimuli [10,11]. Therefore, there is an urgency to better understand the impact of ambient PM_2.5_ exposure on IVF outcomes, particularly live birth, the primary outcome of IVF success.

The association between maternal PM_2.5_ exposure and live birth in IVF patients remains uncertain based on current evidence, including both single-center [[12], [13], [14], [15], [16], [17], [18]] and multicenter [[19], [20], [21], [22]] studies. To our knowledge, only three multicenter studies involving more than ten thousand samples have been reported to date [19,20,22], which limits our understanding of PM_2.5_ effects on live birth after IVF treatment. At present, there is no consensus on hazard windows for PM_2.5_ exposure. The oocyte development phase is commonly recognized as a pre-conceptional stage highly susceptible to exogenous stimuli [23]. However, the existing evidence is highly inconsistent [[12], [13], [14],^16^,^18^,^19^,^22^,[24], [25], [26], [27]]. For instance, a retrospective study involving 15,217 IVF cycles from five Chinese cities found that pre-conceptional PM_2.5_ exposure was associated with poorer reproductive outcomes [19], while two other multicenter analyses did not find any significant associations between pre-conceptional PM_2.5_ exposure and pregnancy outcomes [22,27]. The primary goal of IVF treatment is to achieve a live birth, which depends on the successful progression through pregnancy. However, it remains largely unclear at which stage of pregnancy PM_2.5_-induced live-birth failures predominantly occur. Disaggregating the independent effects of PM_2.5_ on specific pregnancy outcomes could help elucidate the key failure events in the IVF process. Furthermore, the populations most vulnerable to PM_2.5_-related adverse IVF outcomes have not been clearly defined [13,14,18,24,27], largely hindering the implementation of clinical and public practices.

Therefore, in this multicenter study, we analyzed data from 58,637 IVF cycles across six reproductive centers in China using a two-stage analytical framework commonly applied in multicenter epidemiologic studies [28,29]. We aimed to quantify and refine the associations between PM_2.5_ exposure and live birth, and determine the critical exposure windows, key failure events, and vulnerable populations affected by ambient PM_2.5_.

## 2 Materials and methods

### 2.1 Study design and study population

This retrospective study was conducted across six reproductive medicine centers selected based on data availability, medical standards, and regional air quality. To represent a range of exposure, two centers were chosen from regions with good (Fujian and Yunnan), moderate (Liaoning and Shanghai), and poor (Hebei and Shanxi) air quality, respectively (Fig.S1). All centers were affiliated with tertiary (top-tier) hospitals and maintained comparable medical capabilities. For the current analysis, we included women who underwent IVF treatment between January 1, 2016, and December 31, 2021. To avoid potential confounding from repeated cycles within the same patient, only the first IVF cycle for each patient was analyzed. We excluded patients who were over 40 years old, used donor egg or sperm, underwent preimplantation genetic testing, underwent *in vitro* oocyte maturation, had congenital uterine malformations, or had a history of recurrent pregnancy loss (Fig.S2). Medical data were collected, including demographic characteristics, individual medical histories, and relevant risk factors for IVF therapy. The study protocol was approved by the Research Ethics Committee of Shanghai First Maternity and Infant Hospital (KS22298).

### 2.2 Outcome definition

The primary outcome of this study was live birth, defined as the delivery of at least one live-born infant after 22 weeks of gestation. To determine the key failure events contributing to PM_2.5_-associated live-birth failures, we also assessed secondary outcomes, including implantation failure, biochemical pregnancy loss, and miscarriage. Specifically, implantation failure was defined as a serum β-hCG level < 10 mIU/mL approximately 14 days after embryo transfer. Biochemical pregnancy loss was confirmed by the absence of a gestational sac on ultrasound approximately 28 days after embryo transfer in women with successful embryo implantation. Miscarriage was defined as pregnancy loss before 22 weeks of gestation in women who had achieved a clinical pregnancy.

### 2.3 Exposure assessment

**Fig. 1:**
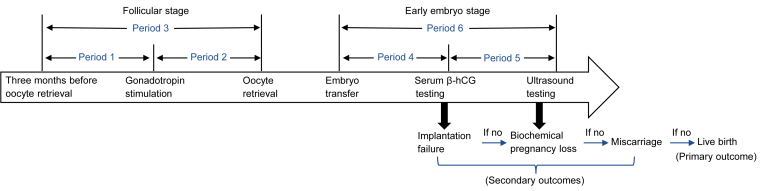
Timeline of *in vitro* fertilization stages and the evaluated exposure windows.

Based on the timeframe of IVF procedures (SI Method 1) and previous literature [12,13,19,20,22,[24], [25], [26],^30^], we defined six exposure windows corresponding to distinct stages of folliculogenesis and embryo development (Fig. 1). Specifically, period 1 (three months before oocyte retrieval to gonadotropin stimulation initiation) encompasses preantral to antral follicle growth [31]. Period 2 (gonadotropin stimulation initiation to oocyte retrieval) represents the ovulation induction phase during which antral follicles mature [31,32]. Period 3 (three months before oocyte retrieval to retrieval) involves the complete oocyte renewal cycle, a well-accepted duration for assessing the chronic effect on oocyte quality and pregnancy outcomes [31,33]. Period 4 (embryo transfer to β-hCG testing) represents the implantation stage, involving embryo–endometrium interaction, trophoblast invasion, and feto-maternal angiogenesis [34,35]. Period 5 (β-hCG testing to ultrasound-confirmed gestation) reflects early embryonic development. Period 6 (embryo transfer to ultrasound-confirmed gestation) comprises the entire early embryogenesis window. The duration of each exposure window was relatively fixed and comparable across six reproductive centers (Table S1).

The average PM_2.5_ concentrations for these defined exposure windows were estimated using a validated satellite-based geospatial prediction model at a resolution of 1 ​km ​× ​1 ​km [36]. In brief, the model used the PM_2.5_ measurements from national monitoring stations as the dependent variable and multiple-source spatiotemporal predictors as independent variables, including satellite aerosol optical depth (AOD), simulated PM_2.5_ concentrations, population density, meteorological data, and land use data. A gap-filling approach with an AOD-model (AOD was available) and a non-AOD model (AOD was unavailable) was applied to generate full-coverage PM_2.5_ predictions. The performance of the random forest model was evaluated with ten-fold cross-validation, yielding a cross-validated R^2^ of 0.86 (daily level) and a root-mean-square error of 14.46 ​μg/m^3^ between predictions and measurements, indicating relatively high predictive accuracy. More details can be found in SI Method 2. Ground-level maximum daily 8-h average ozone (O_3_) and daily nitrogen dioxide (NO_2_) concentrations were also acquired from previously established prediction models with 1-km resolutions [37,38]. Daily ambient temperature data were obtained from the fifth-generation atmospheric reanalysis product (ERA5) at a 10 ​km ​× ​10 ​km resolution [39]. Each participant's residential address was geocoded and then matched to the nearest spatial grid cell, and the average concentrations of pollutants or temperature in the cell were used as exposure estimates for that participant. Daily pollutant predictions were averaged to calculate the mean exposure levels during each exposure window.

### 2.4 Statistical analyses

Descriptive characteristics of patients were summarized as the mean with standard deviation (SD) for continuous variables and count with percentage (%) for categorical variables. To account for potential heterogeneity between centers, we employed a two-stage analytic protocol to assess the associations between ambient PM_2.5_ exposure and live birth. In the first stage, center-specific analyses were performed using binary logistic regression models. The dependent variable was live birth (yes or no), while the independent variable was the average PM_2.5_ concentrations during each exposure window. Covariates listed in Table 1 were included in the main model based on prior literature [14,40,41] and a directed acyclic graph (Fig.S3). Given that our study period included the COVID-19 pandemic onset, we also included a binary variable for the pandemic (Table 1). In the second stage, we performed meta-analysis to pool the center-specific estimates, using a random-effects model. Between-center heterogeneity was quantified using the *I*^2^ statistic.

**Table 1:** Main demographic and clinical characteristics of the baseline population in six study centers.

	Baseline (N ​= ​58,637)	Live birth (N ​= ​24,541)	Non-live birth (N ​= ​34,096)	*P* value^c^
Female age (years)	30.9 (4.0)	30.4 (3.8)	31.3 (4.1)	<0.001
Female BMI (kg/m^2^)	22.4 (3.2)	22.3 (3.2)	22.5 (3.2)	<0.001
Infertility duration (years)	3.90 (2.7)	3.68 (2.5)	3.97 (2.8)	<0.001
Infertility type				0.001
Primary	31,145 (53.1%)	13,224 (53.9%)	17,921 (52.6%)	
Secondary	27,473 (46.9%)	11,307 (46.1%)	16,166 (47.4%)	
Cause of infertility				<0.001
Male-related	9207 (15.7%)	4054 (16.5%)	5153 (15.1%)	
Female-related	36,433 (62.1%)	14,890 (60.7%)	21,543 (63.2%)	
Both-related	9651 (16.5%)	4127 (16.8%)	5524 (16.2%)	
Unexplained	3324 (5.7%)	1462 (6.0%)	1862 (5.5%)	
Gonadotropin dose (IU)	2400 (1010)	2330 (935)	2440 (1050)	<0.001
COH protocols				<0.001
GnRH Antagonist	12,278 (20.9%)	4621 (18.8%)	7657 (22.5%)	
GnRH agonist^a^	44,044 (75.1%)	19,233 (78.4%)	24,811 (72.8%)	
Others^b^	2299 (3.9%)	687 (2.8%)	1612 (4.7%)	
Fertilization methods				0.252
IVF	45,211 (77.1%)	18,914 (77.1%)	26,297 (77.1%)	
ICSI	12,894 (22.0%)	5389 (22.0%)	7505 (22.0%)	
IVF ​+ ​ICSI	492 (0.8%)	224 (0.9%)	268 (0.8%)	
No. of retrieved oocytes	13.3 (8.0)	14.3 (8.1)	12.6 (7.8)	<0.001
No. of high-quality embryos	5.0 (4.1)	5.69 (4.3)	4.54 (3.9)	<0.001
Type of embryo transferred				<0.001
Fresh	34,960 (59.6%)	14,116 (57.5%)	20,844 (61.1%)	
Frozen	23,670 (40.4%)	10,423 (42.5%)	13,247 (38.9%)	
No. of days of embryo culture				<0.001
2	177 (0.3%)	65 (0.3%)	112 (0.3%)	
3	44,003 (75.0%)	17,508 (71.3%)	26,495 (77.7%)	
4	990 (1.7%)	337 (1.4%)	653 (1.9%)	
5	12,178 (20.8%)	6089 (24.8%)	6089 (17.9%)	
6	1258 (2.1%)	529 (2.2%)	729 (2.1%)	
7	16 (0.0%)	7 (0.0%)	9 (0.0%)	
No. of embryo transferred				<0.001
1	13,726 (23.4%)	4466 (18.2%)	9260 (27.2%)	
2	44,803 (76.4%)	20,046 (81.7%)	24,757 (72.6%)	
3	50 (0.1%)	13 (0.1%)	37 (0.1%)	
Endometrial thickness (mm)	9.8 (2.2)	9.9 (2.3)	9.7 (2.2)	<0.001
Season of embryo transfer				0.098
Spring	14,703 (25.1%)	6160 (25.1%)	8543 (25.1%)	
Summer	17,544 (29.9%)	7313 (29.8%)	10,231 (30.0%)	
Autumn	15,719 (26.8%)	6826 (27.8%)	8893 (26.1%)	
Winter	10,671 (18.2%)	4242 (17.3%)	6429 (18.9%)	
COVID-19 pandemic period				<0.001
No	40,395 (68.9%)	16,367 (66.7%)	24,028 (70.5%)	
Yes	18,242 (31.1%)	8174 (33.3%)	10,068 (29.5%)	

Continuous variables were presented with mean and standard deviation (SD), categorical variables were presented with count and percentage (%). Spring, March–May; Summer, June–August; Autumn, September–November; Winter, December–February. The COVID-19 pandemic period refers to whether any reproductive outcomes occur after January 1, 2020. BMI, body mass index; COH, controlled ovarian hyperstimulation; IVF, in vitro fertilization; ICSI, intracytoplasmic sperm injection; No., number.

a GnRH agonist includes long agonist, ultra-long agonist, short agonist, and ultra-short agonist.

b Other COH protocols included progestin-primed ovarian stimulation protocol (PPOS), luteal-phase ovarian stimulation, and mild stimulation protocols.

c *P* value was derived from a *t*-test or a chi-square test for the difference between the participants with live birth and without.

To examine the robustness of the associations between PM_2.5_ exposure and live birth, several sensitivity analyses were conducted using the same two-stage analytic protocol. First, we limited the analyses to local patients residing in the province or municipality where the reproductive centers are located to minimize potential exposure misclassification. Second, we compared the associations between PM_2.5_ exposure and live birth with and without adjustments for couples' smoking status, education levels, and female occupational exposures, within centers having complete records, to address their potential confounding. Third, we constructed two-pollutant models by separately incorporating NO_2_ and O_3_ concentrations into the main model, using the same exposure window as for PM_2.5_. Fourth, we used a natural spline with three degrees of freedom to control the potential nonlinear and acute effects of the average temperature in three days prior to pregnancy outcome.

Then, we conducted analyses to evaluate the impact of PM_2.5_ exposure on secondary outcomes, including implantation failure, biochemical pregnancy loss, and miscarriage, with parameters consistent with the primary outcome models. Finally, we performed stratified analyses based on female age (<31 years and ≥31 years), number of oocytes retrieved (<10 oocytes, 10−15 oocytes, and >15 oocytes), high-quality embryo rate (>60% and <30%) [42], type of embryos transferred (fresh and frozen), and number of embryos transferred (one and two), respectively. The number of oocytes retrieved was used to categorize ovarian responders: poor and suboptimal (<10 oocytes), normal (10−15 oocytes), and excessive (>15 oocytes) [40,43]. High-quality embryo rate was calculated as the number of high-quality embryos divided by the number of normal fertilizations. In addition, we performed a stratified analysis by the season of oocytes retrieved.

All statistical analyses were conducted using R software (version 4.0.3), with the “glm” function for logistic regressions and the “meta” package for meta-analysis. The estimates were reported as odds ratios (ORs) and 95% CIs for pregnancy outcomes per 10 ​μg/m^3^ increase in PM_2.5_ concentration. The statistical tests were two-sided, and *P* ​< ​0.05 was considered statistically significant.

## 3 Results

A total of 58,637 patients were included in the analysis. The demographic and clinical characteristics of the study population are summarized in Table 1. The average age of the female patients was 30.9 years, with an average body mass index of 22.4 ​kg/m^2^ and an infertility duration of 3.9 years. Female-related factor was the major cause (62.1%) of infertility. Most of the patients received GnRH agonist treatment (75.1%), underwent conventional IVF for fertilization (77.1%), and had double embryo transfer (76.4%). Significant differences between the live birth and non-live birth groups were observed for ovarian function (e.g., female age and number of retrieved oocytes), infertility characteristics (cause, type, and duration), and IVF treatment protocols (e.g., embryo type and number transferred). Additional detailed information from each center is presented in Tables S2−S7.

**Fig. 2:**
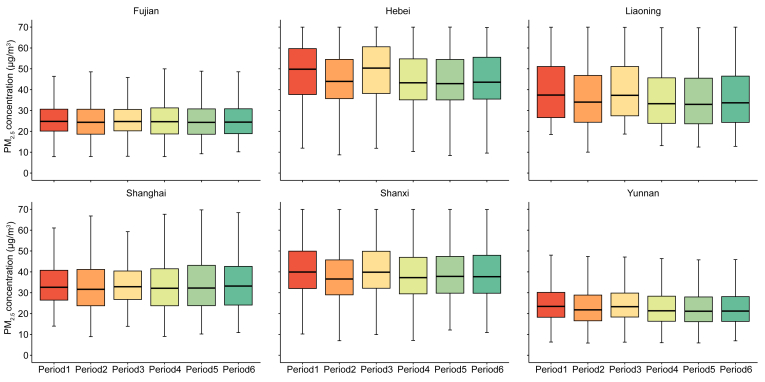
Box plot of fine particulate matter during six exposure periods among the participants undergoing *in vitro* fertilization in six study centers. The box represents the 25th and 75th percentiles range, and the whiskers represent 1.5× ​the interquartile range.

Fig. 2 presents the distribution of the average PM_2.5_ concentrations across six exposure windows for each center. The mean PM_2.5_ concentrations varied considerably between centers, with the lowest levels in Yunnan (23.19–24.77 ​μg/m^3^) and the highest levels in Hebei (59.24–63.86 ​μg/m^3^) (Table S8), indicating the potential for heterogeneous effects of PM_2.5_ exposure. Within-center variations across exposure windows were relatively small, such as in Fujian, where the PM_2.5_ concentration ranged between 25.88 ​μg/m^3^ and 26.33 ​μg/m^3^ (Table S8).

Table 2 shows both the center-specific and pooled associations between PM_2.5_ exposure and live birth. We observed that PM_2.5_ exposure was negatively associated with live birth in most reproductive centers. For pooled analyses, significant negative associations were observed between PM_2.5_ exposure during follicular stages (Period 1–Period 3) and live birth (Table 2). The OR (95% CI) for live birth per 10 ​μg/m^3^ increment in PM_2.5_ concentrations during Period 1–Period 3 were 0.967 (0.939, 0.996), 0.978 (0.958, 0.998), and 0.966 (0.938, 0.995), respectively. In contrast, the associations between PM_2.5_ exposures during early embryo stages (Period 4–Period 6) and live birth were negative but not statistically significant. The heterogeneity in the associations across centers was moderate, with the *I*^2^ statistic ranging from 43.5% to 56.8%.

**Table 2:** Odds ratios and 95% confidence intervals for live birth per 10 ​μg/m^3^ increase in PM_2.5_ for *in vitro* fertilization cycles.

	Period 1	Period 2	Period 3	Period 4	Period 5	Period 6
Pooled	0.967 (0.939, 0.996)	0.978 (0.958, 0.998)	0.966 (0.938, 0.995)	0.993 (0.971, 1.015)	0.989 (0.959, 1.018)	0.986 (0.957, 1.016)
Center-specific						
Fujian	0.936 (0.884, 0.991)	0.935 (0.892, 0.980)	0.930 (0.878, 0.986)	0.938 (0.894, 0.983)	0.918 (0.849, 0.992)	0.922 (0.851, 0.999)
Hebei	0.968 (0.951, 0.985)	0.977 (0.964, 0.990)	0.966 (0.950, 0.984)	0.983 (0.968, 0.999)	0.996 (0.970, 1.023)	0.985 (0.959, 1.013)
Liaoning	0.905 (0.847, 0.968)	0.970 (0.929, 1.012)	0.910 (0.850, 0.974)	0.997 (0.951, 1.045)	0.964 (0.896, 1.036)	0.965 (0.898, 1.038)
Shanghai	1.030 (0.942, 1.126)	1.000 (0.945, 1.058)	1.025 (0.936, 1.122)	1.029 (0.970, 1.091)	0.994 (0.913, 1.084)	0.998 (0.906, 1.099)
Shanxi	1.006 (0.967, 1.047)	1.016 (0.985, 1.047)	1.009 (0.969, 1.050)	1.022 (0.990, 1.055)	1.043 (0.994, 1.095)	1.048 (0.996, 1.102)
Yunnan	0.966 (0.937, 0.995)	0.967 (0.943, 0.992)	0.962 (0.933, 0.993)	0.995 (0.971, 1.020)	0.979 (0.940, 1.020)	0.977 (0.937, 1.018)

The definition of exposure periods refers to Fig. 1.

Sensitivity analyses were performed for Period 1–Period 3 due to the significant associations observed. The associations between PM_2.5_ and live birth remained stable when excluding patients whose residential addresses were outside the province or municipality of the center (Table S9). Additionally, adjusting for factors such as smoking, education levels, and female occupation did not substantially alter the associations between PM_2.5_ and live birth (Table S9). Comparison between adjusted and unadjusted results only showed minimal variations. The associations between PM_2.5_ during the follicular growth stages and live birth also remained robust with adjustment for O_3_, NO_2,_ and temperature (Table S9).

**Fig. 3:**
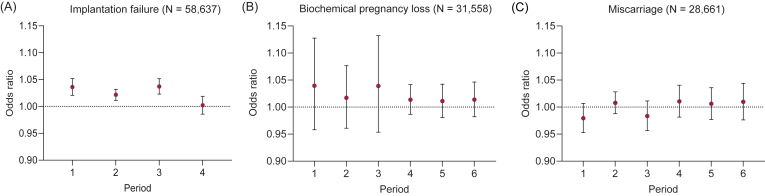
Pooled odds ratios and 95% confidence intervals for implantation failure (A), biochemical pregnancy loss (B), and miscarriage (C) per 10 ​μg/m^3^ increase in PM_2.5_ concentration among participants undergoing *in vitro* fertilization. The definition of exposure periods refers to Fig. 1.

Fig. 3 presents the effects of PM_2.5_ on implantation failure, biochemical pregnancy loss, and miscarriage. For the overall population, PM_2.5_ exposure during the follicular stage (Period 1–Period 3) was significantly and positively associated with implantation failure. In women with successful embryo implantation, the associations between PM_2.5_ exposure and biochemical pregnancy loss were positive but not statistically significant. No substantial associations were observed between PM_2.5_ and miscarriage as pregnancy progressed.

**Fig. 4:**
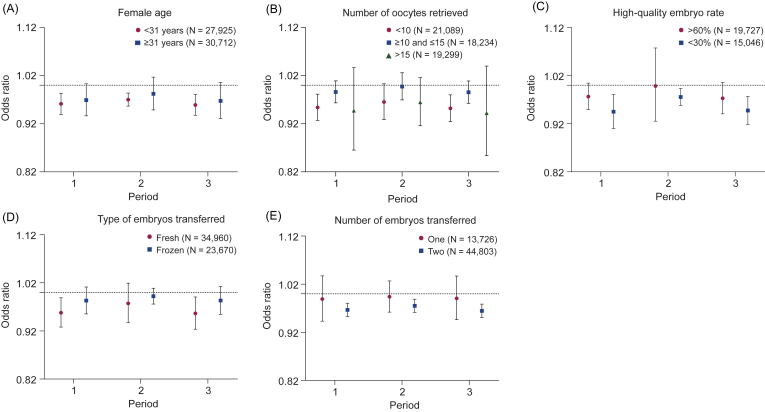
Pooled odds ratios and 95% confidence intervals for live birth per 10 ​μg/m^3^ increase in PM_2.5_ concentration, stratified by female age (A), number of oocytes retrieved (B), high-quality embryo rate (C), type of embryos transferred (D), and number of embryos transferred (E). The definition of exposure periods refers to Fig. 1. The high-quality embryo rate is calculated by dividing the number of high-quality embryos (numerator) by the number of normal fertilizations (denominator) per patient.

Given the significant and robust results, we conducted stratified analyses only for PM_2.5_ during Period 1–Period 3. As depicted in Fig. 4, we observed that the associations between PM_2.5_ exposures and live birth were more pronounced in females with poor and suboptimal ovarian response (<10 oocytes retrieved), women with a high-quality embryo rate <30%, and those who underwent a fresh transfer or a double embryo transfer. In addition, we found that the PM_2.5_ exposure was associated with lower odds of live birth in winter than in other seasons (Table S10).

## 4 Discussion

This multicenter, population-based study demonstrated that ambient PM_2.5_ exposure during the follicular growth stages was significantly associated with decreased odds of live birth in females undergoing IVF treatment, and this PM_2.5_-induced live birth failure mainly occurred during the embryo implantation period, i.e., implantation failures. Furthermore, we identified that women with diminished ovarian response, poor embryo quality, and those undergoing a fresh or double-embryo transfer were more susceptible to the negative effects of PM_2.5_ exposure.

The multicenter data integration and the large-scale sample size strengthen the reliability and generalizability of our findings, that exposure to PM_2.5_ during the follicular growth stage is a risk factor for live birth. This is aligned with results from several single-center and multicenter retrospective studies [12,13,18,19]. However, previous studies have rarely addressed the potential heterogeneity between centers or locations, which may overlook variability in true estimates [29,44]. In our study, moderate heterogeneity was observed across centers, with *I*^2^ statistics ranging from 43.5% to 56.8%. By combining data across centers using a two-stage analytic protocol, we were able to provide more compelling evidence of the associations between PM_2.5_ and live birth. These associations also remain robust in sensitivity analyses. The associations of PM_2.5_ in the follicular growth stages with live birth could be explained by the potential detriment of PM_2.5_ to oocyte quality and embryo quality [18,45,46], as we correspondingly found stronger detrimental impacts in the women with diminished ovarian response and poor embryo quality. The possibility is also supported by experimental studies. For instance, exposing female mice to PM_2.5_ with a high average concentration of 115.6 ​μg/m^3^ increased oocyte degeneration rate and decreased 2-cell embryo formation rate compared to mice exposed to filtered air [8]. In this study, PM_2.5_ exposure during the oocyte development stage, particularly the preantral-antral follicle transition stage, was associated with lower odds of live birth. These findings support the importance of considering interventions such as wearing a mask outdoors to minimize ambient PM_2.5_ exposure during the follicular stages.

The conditional definition of pregnancy outcomes allowed us to identify the key failure events contributing to live-birth failures induced by PM_2.5_ and the possible lag effects of PM_2.5_ across the IVF process. In our research, we found that maternal PM_2.5_ exposure during follicular stages (Period 1–Period 3) was primarily associated with elevated odds of implantation failures. Although the exact mechanisms underlying these associations remain unclear, a possible explanation is that PM_2.5_ exposure during follicular stages may disrupt the uterine microenvironment, impairing embryo implantation via supraphysiological reactive oxygen species [47,48]. In addition, previous evidence has demonstrated that particles may affect the expression of endometrial receptive markers, thereby affecting embryo implantation [49,50], suggesting that endocrine disruption is another possible mechanism. We also observed that the adverse effects of PM_2.5_ exposure in specific exposure windows tended to diminish over time as the IVF cycle progressed. This might reflect the depletion of vulnerable patients during IVF, as only those who successfully progress through earlier stages remain in later stages. Similar depletion was observed in a prospective clinical study by Gaskins et al., who reported that the time-varying effects of air pollution varied over the course of the IVF cycle [51]. In summary, our results further refined the effects of PM_2.5_ on live birth and provided a primary point for attention, e.g., implantation failure, when evaluating the effects and mechanisms of maternal PM_2.5_ on pregnancy.

Identifying vulnerable subgroups is crucial to minimizing the adverse effects of PM_2.5_ exposure on pregnancy. Our studies showed that women with poor ovarian response and embryo quality exhibited lower odds of live birth in relation to PM_2.5_ exposure during follicular stages, suggesting that individuals with poorer maternal health might be a vulnerable subgroup. This finding is biologically plausible and was supported by some animal evidence on PM_2.5_-attributed oocyte impairments [8,52]. We also observed that women who underwent a fresh embryo transfer might be more susceptible to the negative impacts of PM_2.5_ exposure. This may be due to the high steroid environment in women with fresh embryo transfers, which could be associated with ovarian stimulation procedures several days before and exacerbated by the ongoing effects of PM_2.5_ in former periods [53,54]. In contrast, women who underwent frozen embryo transfer have a long embryo-freezing duration, which may benefit them from a more stable uterine environment and diluent PM_2.5_ effects from previous stages. Furthermore, PM_2.5_ during the follicular stages was associated prominently with live birth in women who underwent double embryo transfers. This effect modification was in accordance with several previous studies [13,18], and might be ascribed to the additive adverse impact of PM_2.5_ on each follicle, disrupting the uterine environment. In addition, we also found that PM_2.5_-associated live birth odds may be lower in winter. The role of seasonality on IVF progress has not been well established yet, but this phenomenon can be attributed to elevated ambient PM_2.5_ levels in winter. Some studies reported the same findings and suggested this modification may be further related to vitamin D, a cornerstone of reproductive health, which typically reaches lower in winter [55,56]. Overall, our results suggested several possible effect modifiers, and women undergoing one embryo or frozen embryo transfer may be less affected by the ambient PM_2.5_, providing a consideration for clinical practice.

One key strength of this study is the extensive sample size and the integration of data from multiple centers, which provided stronger statistical power to assess the associations between ambient PM_2.5_ exposure and live birth. The two-stage analytic protocol accounted for potential heterogeneity in the estimates across centers, thereby improving the generalizability of our results. Additionally, by defining progressive pregnancy outcomes through the IVF procedure, we were able to pinpoint the earliest stages of pregnancy as the main time period affected by PM_2.5_ exposure, providing clues for understanding the effects of ambient PM_2.5_ across IVF treatment. Our study also has several limitations. First, our study relied on model-predicted PM_2.5_ concentrations, which may introduce exposure misclassification. However, such misclassification is likely to be random, potentially biasing the results towards the null. Then, there were some missing data related to smoking, occupation, and education level, although we adjusted for these variables in the sensitivity analyses, minor differences may exist for whole-data adjustment. Third, though a range of covariates were considered, we failed to include some indicators, such as sleep quality, drinking status, and sperm quality, for adjustment. In addition, we acknowledge that selective bias may exist in representing the overall IVF population in China, as we only selected six reproductive centers. Finally, given the retrospective nature of this study, it is difficult to establish causality, and further experimental studies are needed to explore the underlying mechanisms of PM_2.5_ exposure on pregnancy outcomes.

## 5 Conclusion

This large-scale population-based study provides evidence linking ambient PM_2.5_ exposure during the follicular growth stages to the decreased odds of live birth in women undergoing IVF treatments. Our results suggested that the stage of follicular growth was the critical exposure window, and embryo implantation failure was the key event leading to reduced live birth due to PM_2.5_ exposure. Women with diminished ovarian response or poor embryo quality, and those undergoing fresh embryo or double embryo transfers, were more susceptible to these effects. These results offer valuable insights for clinical and public health strategies aimed at reducing the risks associated with PM_2.5_ exposure.

## CRediT authorship contribution statement

**Miaoxin Chen:** Writing – review & editing, Writing – original draft, Visualization, Validation, Methodology, Investigation, Funding acquisition, Data curation, Conceptualization. **Ying Fang:** Writing – review & editing, Writing – original draft, Visualization, Validation, Methodology, Formal analysis, Data curation, Conceptualization. **Yunxiu Li:** Writing – review & editing, Validation, Methodology, Data curation, Conceptualization. **Guimin Hao:** Writing – review & editing, Validation, Methodology, Data curation, Conceptualization. **Xueqing Wu:** Writing – review & editing, Validation, Methodology, Data curation, Conceptualization. **Yan Sun:** Writing – review & editing, Validation, Methodology, Data curation, Conceptualization. **Jichun Tan:** Writing – review & editing, Validation, Methodology, Data curation, Conceptualization. **Yue Niu:** Writing – review & editing, Writing – original draft, Visualization, Validation, Methodology, Formal analysis, Data curation, Conceptualization. **Xinyi Du:** Writing – review & editing, Investigation, Data curation, Conceptualization. **Yonggang Li:** Writing – review & editing, Investigation, Data curation, Conceptualization. **Zhuoye Luo:** Writing – review & editing, Investigation, Data curation, Conceptualization. **Fen Hu:** Writing – review & editing, Investigation, Data curation, Conceptualization. **Yuehong Li:** Writing – review & editing, Investigation, Data curation, Conceptualization. **Shanshan Wu:** Writing – review & editing, Investigation, Data curation, Conceptualization. **Yingying Yang:** Writing – review & editing, Investigation, Data curation. **Orhan Bukulmez:** Writing – review & editing, Validation, Methodology, Conceptualization. **Yeung William Shu-Biu:** Writing – review & editing, Validation, Methodology, Conceptualization. **Robert J. Norman:** Writing – review & editing, Validation, Methodology, Conceptualization. **Haidong Kan:** Writing – review & editing, Supervision, Resources, Project administration, Methodology, Funding acquisition, Conceptualization. **Xiaoming Teng:** Writing – review & editing, Supervision, Resources, Project administration, Methodology, Funding acquisition, Conceptualization.

## Declaration of competing interests

The authors have declared no conflicts of interest.

## References

[1] Eskew A.M., Jungheim E.S. (2017). A history of developments to improve in vitro fertilization. Mo. Med..

[2] Fauser B.C.J.M. (2019). Towards the global coverage of a unified registry of IVF outcomes. Reprod. Biomed. Online.

[3] Bekkar B., Pacheco S., Basu R., DeNicola N. (2020). Association of air pollution and heat exposure with preterm birth, low birth weight, and stillbirth in the US. JAMA Netw. Open.

[4] Song S., Gao Z., Zhang X., Zhao X., Chang H., Zhang J. (2023). Ambient fine particulate matter and pregnancy outcomes: an umbrella review. Environ. Res..

[5] Wang H., Zhu Z., Benmarhnia T., Chen X., Jalaludin B., Wulayin M. (2024). Estimation of couple fecundity in the general population and the association with monthly time-varying ambient particulate matter exposure in low- and middle-income countries: a population-based multi-center epidemiological study. Environ. Int..

[6] Gong C., Chu M., Yang J., Gong X., Han B., Chen L. (2022). Ambient fine particulate matter exposures and human early placental inflammation. Environ. Pollut..

[7] Zhao Y., Wang P., Zhou Y., Xia B., Zhu Q., Ge W. (2021). Prenatal fine particulate matter exposure, placental DNA methylation changes, and fetal growth. Environ. Int..

[8] Guo Y., Cao Z., Jiao X., Bai D., Zhang Y., Hua J. (2021). Pre-pregnancy exposure to fine particulate matter (PM_2.5_) increases reactive oxygen species production in oocytes and decrease litter size and weight in mice. Environ. Pollut..

[9] Nachman R.M., Mao G., Zhang X., Hong X., Chen Z., Soria C.S. (2016). Intrauterine inflammation and maternal exposure to ambient PM_2.5_ during preconception and specific periods of pregnancy: the Boston birth cohort. Environ. Health Perspect..

[10] Perin P.M., Maluf M., Czeresnia C.E., Nicolosi Foltran Januário D.A., Nascimento Saldiva P.H. (2010). Effects of exposure to high levels of particulate air pollution during the follicular phase of the conception cycle on pregnancy outcome in couples undergoing in vitro fertilization and embryo transfer. Fertil. Steril..

[11] Woo I., Hindoyan R., Landay M., Ho J., Ingles S.A., McGinnis L.K. (2017). Perinatal outcomes after natural conception versus in vitro fertilization (IVF) in gestational surrogates: a model to evaluate IVF treatment versus maternal effects. Fertil. Steril..

[12] Dai W., Shi H., Bu Z., Yu Y., Sun Z., Hu L. (2021). Ambient air pollutant exposure and in vitro fertilization treatment outcomes in Zhengzhou, China. Ecotoxicol. Environ. Saf..

[13] Zhang C., Yao N., Lu Y., Ni J., Liu X., Zhou J. (2022). Ambient air pollution on fecundity and live birth in women undergoing assisted reproductive technology in the Yangtze river Delta of China. Environ. Int..

[14] Shi W., Sun C., Chen Q., Ye M., Niu J., Meng Z. (2021). Association between ambient air pollution and pregnancy outcomes in patients undergoing in vitro fertilization in Shanghai, China: a retrospective cohort study. Environ. Int..

[15] Wang Y., Qiu Y., Huang B., Du J., Liu L., Jiang T. (2023). Association between PM_2.5_ exposure and the outcomes of ART treatment: a prospective birth cohort study. Sci. Total Environ..

[16] Zhang X., Wu S., Lu Y., Qi J., Li X., Gao S. (2024). Association of ambient PM_2.5_ and its components with in vitro fertilization outcomes: the modifying role of maternal dietary patterns. Ecotoxicol. Environ. Saf..

[17] Huang K., Hu M., Zhang Z., Li Z., Hu C., Bai S. (2025). Associations of ambient air pollutants with pregnancy outcomes in women undergoing assisted reproductive technology and the mediating role of ovarian reserve: a longitudinal study in eastern China. Sci. Total Environ..

[18] Huang K., Zhang Z., Hu M., Zhao J., Li Z., Hu C. (2025). Association of specific PM_2.5_ chemical constituents and ozone exposure with pregnancy outcomes in women undergoing assisted reproductive technology treatment in central China. Int. J. Hyg Environ. Health.

[19] Wu S., Zhang Y., Hao G., Chen X., Wu X., Ren H. (2023). Interaction of air pollution and meteorological factors on IVF outcomes: a multicenter study in China. Ecotoxicol. Environ. Saf..

[20] Boulet S.L., Zhou Y., Shriber J., Kissin D.M., Strosnider H., Shin M. (2019). Ambient air pollution and in vitro fertilization treatment outcomes. Hum. Reprod..

[21] Quraishi S.M., Lin P.C., Richter K.S., Hinckley M.D., Yee B., Neal-Perry G. (2019). Ambient air pollution exposure and fecundability in women undergoing in vitro fertilization. Environ. Epidemiol..

[22] Wu S., Zhang Y., Wu X., Hao G., Ren H., Qiu J. (2021). Association between exposure to ambient air pollutants and the outcomes of in vitro fertilization treatment: a multicenter retrospective study. Environ. Int..

[23] Fleming T.P., Watkins A.J., Velazquez M.A., Mathers J.C., Prentice A.M., Stephenson J. (2018). Origins of lifetime health around the time of conception: causes and consequences. Lancet.

[24] Jin H.-x., Guo Y.-h., Song W.-y., Li G., Liu Y., Shi S.-l. (2022). Effect of ambient air pollutants on in vitro fertilization-embryo transfer pregnancy outcome in Zhengzhou, China. Environ. Toxicol. Pharmacol..

[25] Qiu J., Dong M., Zhou F., Li P., Kong L., Tan J. (2019). Associations between ambient air pollution and pregnancy rate in women who underwent in vitro fertilization in Shenyang, China. Reprod. Toxicol..

[26] Zeng X., Jin S., Chen X., Qiu Y. (2020). Association between ambient air pollution and pregnancy outcomes in patients undergoing in vitro fertilization in Chengdu, China: a retrospective study. Environ. Res..

[27] Lan C., Guan Y., Luo H., Ma X., Yang Y., Bao H. (2024). Observed effects on very early pregnancy linked to ambient PM_2.5_ exposure in China among women undergoing in vitro fertilization-embryo transfer. Environ. Health.

[28] Liu C., Chen R., Sera F., Vicedo-Cabrera A.M., Guo Y., Tong S. (2019). Ambient particulate air pollution and daily mortality in 652 cities. N. Engl. J. Med..

[29] Chen R., Yin P., Meng X., Liu C., Wang L., Xu X. (2017). Fine particulate air pollution and daily mortality. A nationwide analysis in 272 Chinese cities. Am. J. Respir. Crit. Care Med..

[30] Li L., Zhou L., Feng T., Hao G., Yang S., Wang N. (2020). Ambient air pollution exposed during preantral-antral follicle transition stage was sensitive to associate with clinical pregnancy for women receiving IVF. Environ. Pollut..

[31] Oktem O., Urman B. (2010). Understanding follicle growth in vivo. Hum. Reprod..

[32] Liu J., Dai Y., Yuan J., Li R., Hu Y., Su Y. (2023). Does exposure to air pollution during different time windows affect pregnancy outcomes of in vitro fertilization treatment? A systematic review and meta-analysis. Chemosphere.

[33] Gougeon A. (1986). Dynamics of follicular growth in the human: a model from preliminary results. Hum. Reprod..

[34] Annan (2013). Biochemical pregnancy during assisted conception: a little bit pregnant. J. Clin. Med. Res..

[35] Herr F., Baal N., Reisinger K., Lorenz A., McKinnon T., Preissner K.T. (2007). hCG in the regulation of placental angiogenesis. Results of an in vitro study. Placenta.

[36] Shi S., Wang W., Li X., Xu C., Lei J., Jiang Y. (2023). Evolution in disparity of PM_2.5_ pollution in China. Eco-Environ. Health.

[37] Meng X., Wang W., Shi S., Zhu S., Wang P., Chen R. (2022). Evaluating the spatiotemporal ozone characteristics with high-resolution predictions in mainland China, 2013–2019. Environ. Pollut..

[38] Li X., Wang P., Wang W., Zhang H., Shi S., Xue T. (2023). Mortality burden due to ambient nitrogen dioxide pollution in China: application of high-resolution models. Environ. Int..

[39] Muñoz-Sabater J., Dutra E., Agustí-Panareda A., Albergel C., Arduini G., Balsamo G. (2021). ERA5-Land: a state-of-the-art global reanalysis dataset for land applications. Earth Syst. Sci. Data.

[40] Wu S., Hao G., Zhang Y., Chen X., Ren H., Fan Y. (2022). Poor ovarian response is associated with air pollutants: a multicentre study in China. EBioMedicine.

[41] Shingshetty L., Cameron N.J., McLernon D.J., Bhattacharya S. (2024). Predictors of success after in vitro fertilization. Fertil. Steril..

[42] Xu J., Wang Q., Jiao X., Kong P., Chen S., Yang W. (2025). Association between perfluorooctanoic acid-related poor embryo quality and metabolite alterations in human follicular fluid during IVF:A Cohort study. Environ. Health Perspect..

[43] Polyzos N.P., Sunkara S.K. (2015). Sub-optimal responders following controlled ovarian stimulation: an overlooked group?. Hum. Reprod..

[44] Bell M.L., Dominici F., Samet J.M. (2005). A meta-analysis of time-series studies of ozone and mortality with comparison to the national morbidity, mortality, and air pollution study. Epidemiology.

[45] Pang L., Yu W., Lv J., Dou Y., Zhao H., Li S. (2023). Air pollution exposure and ovarian reserve impairment in Shandong province, China: the effects of particulate matter size and exposure window. Environ. Res..

[46] Fang L., Ma C., Ma Y., Zhao H., Peng Y., Wang G. (2023). Associations of long-term exposure to air pollution and green space with reproductive hormones among women undergoing assisted reproductive technology: a longitudinal study. Sci. Total Environ..

[47] Lin J., Wang L. (2021). Oxidative stress in oocytes and embryo development: implications for In vitro systems. Antioxidants Redox Signal..

[48] Combelles C.M.H., Gupta S., Agarwal A. (2009). Could oxidative stress influence the in-vitro maturation of oocytes?. Reprod. Biomed. Online.

[49] Liang Y., Shuai Q., Wang Y., Jin S., Feng Z., Chen B. (2021). 1-Nitropyrene exposure impairs embryo implantation through disrupting endometrial receptivity genes expression and producing excessive ROS. Ecotoxicol. Environ. Saf..

[50] de Castro K.R., Almeida G.H.D.R., Matsuda M., de Paula Vieira R., Martins M.G., Rici R.E.G. (2024). Exposure to urban ambient particles (PM_2.5_) before pregnancy affects the expression of endometrial receptive markers to embryo implantation in mice: preliminary results. Tissue Cell.

[51] Gaskins A.J., Fong K.C., Abu Awad Y., Di Q., Mínguez-Alarcón L., Chavarro J.E. (2019). Time-varying exposure to air pollution and outcomes of in vitro fertilization among couples from a fertility clinic. Environ. Health Perspect..

[52] Liao B.-Q., Liu C.-B., Xie S.-J., Liu Y., Deng Y.-B., He S.-W. (2020). Effects of fine particulate matter (PM_2.5_) on ovarian function and embryo quality in mice. Environ. Int..

[53] Shi Y., Sun Y., Hao C., Zhang H., Wei D., Zhang Y. (2018). Transfer of fresh versus frozen embryos in ovulatory women. N. Engl. J. Med..

[54] Weinerman R., Mainigi M. (2014). Why we should transfer frozen instead of fresh embryos: the translational rationale. Fertil. Steril..

[55] Li F., Duan X., Li M., Gao Y., Kang Y., Zheng W. (2025). Environmental pollution and human fertility: investigating the relationship between PM_2.5_ exposure and assisted reproductive technology outcomes. BMC Public Health.

[56] Zhi X., Chen Y. (2020). Roles of vitamin D in reproductive systems and assisted reproductive technology. Endocrinology.

