# Supplementary material for: Maternal exposure to fine particulate matter and pregnancy outcomes in women undergoing in vitro fertilization: A multicenter retrospective study
Source: Eco Environ Health. 2025 Oct 15;4(4):100192. doi: 10.1016/j.eehl.2025.100192 (PMC12766417; doi:10.1016/j.eehl.2025.100192)
Supplement: Multimedia component 1 [file mmc1.docx]

**Supplementary Materials**

**Maternal exposure to fine particulate matter and pregnancy outcomes in women undergoing *in vitro* fertilization: A multicenter retrospective study**

**SI Method 1.** *In vitro* fertilization procedures

**SI Method 2.** Air pollutant exposure estimations

**Table S1.** Average duration and standard deviations for each exposure window

**Table S2.** Demographic and clinical characteristics of the study population in Fujian

**Table S3.** Demographic and clinical characteristics of the study population in Hebei

**Table S4.** Demographic and clinical characteristics of the study population in Liaoning

**Table S5.** Demographic and clinical characteristics of the study population in Shanghai

**Table S6.** Demographic and clinical characteristics of the study population in Shanxi

**Table S7.** Demographic and clinical characteristics of the study population in Yunnan

**Table S8.** The distributions of PM_2.5_ during six exposure periods among the participants undergoing *in vitro* fertilization in six study centers

**Table S9.** Sensitivity analyses for associations between ambient PM_2.5_ and live birth among patients undergoing *in vitro* fertilization

**Table S10.** Pooled odds ratios and 95% confidence intervals for live birth, stratified by season of oocyte retrieval

**Fig.S1.** The geographical distribution of study patients

**Fig.S2.** Flowchart of *in vitro* cycles included in this study

**Fig.S3.** Directed Acyclic Graph for the associations between fine particulate pollution and live birth

**SI Methods 1**

The overall *in vitro* fertilization (IVF) procedures generally include four stages: controlled ovarian hyperstimulation (COH), oocyte retrieval, embryo transfer, and luteal phase support. All patients were followed up throughout the whole IVF cycle until pregnancy failure or delivery. The details of IVF procedures have been published previously [1]. Briefly, patients underwent individual-based COH from day 2 or day 3 of the menstrual cycle. COH protocols included gonadotropin-releasing hormone (GnRH)-agonist or GnRH antagonist (-ant) protocol or other stimulation protocols (i.e., mild stimulation protocol), and their adoption was decided by physicians. The follicular growth of patients was monitored by the level of serum estradiol and ultrasonography during ovarian stimulation. Ovulation was triggered by injection of hCG (Ovidrel; Merck Serono) when at least two follicles reached a diameter of 18mm. Oocyte retrieval was conducted 34–36 h after hCG injection. In women with hyper-response (≥15 follicles ≥12 mm), 0.2 mg Triptorelin or 4000 IU of hCG was administered. Semen preparation, conventional IVF, ICSI, embryo culture, and embryo assessment were performed by standard routines at study centers. Embryos were graded as good quality on day 3 if they developed from 2PN embryos with 7–10 blastomeres, <20% anucleate fragments, and no apparent morphologic abnormalities. For patients who received fresh embryo transfer, cleavage-stage embryos were transferred on day 2 or 3, and blastocysts were transferred on day 5 following oocyte retrieval guided by transabdominal ultrasound; and surplus embryos were frozen. Similarly, for women with hyper-response, all embryos were frozen on day 2-3, or day 5-6 after retrieval. For patients who underwent frozen embryo transfer, endometrial preparation protocols, including hormone replacement treatment and natural cycles, and transfer procedures were performed by standard routines at the study center. Luteal-phase support was started on the day of retrieval, and for women with a positive hCG test, luteal-phase support was continued until 10 weeks of gestation.

.

**SI Methods 2**

The methodology of predicting PM_2.5_ concentrations has been published in our previous studies [2, 3] and was summarized here. The PM_2.5_ model was established using a gap-filling approach with random forest algorithms to associate ground PM_2.5_ measurements with multiple predictors, including aerosol optical depth (AOD), stimulated PM_2.5_ concentrations, population density, meteorological parameters, and land use variables.

**Data sources and preprocessing:** Hourly ground PM_2.5_ measurements from national stations covering mainland China were sourced from the National Air Quality Monitoring Network (http://www.cnemc.cn/). Multi-Angle Implementation of Atmospheric Correction AOD with spatial 1-km resolution from satellites Aqua and Terra was downloaded at the Earth Data portal (https://search.earthdata.nasa.gov). To process the high-missing AOD data, the linear regression models between satellites (AODAuqa_mean and AODTerra_mean) at the daily level were first employed to predict the missing AOD from one satellite at grid-days with the other. Daily simulated PM_2.5_ concentrations from the Modern-Era Retrospective analysis for Research and Applications, Version 2 (MERRA-2) were collected. The simulated PM_2.5_ values were then interpolated by the inverse distance weighted (IDW) method to 1-km grid cells. Meteorological data, comprising air temperature (at 2 m), wind (at 10 km), relative humidity (at 2 m), surface pressure, and planetary boundary layer height, were downscaled by IDW to 1-km grid cells. Other land use (Elevation and The Normalized Difference Vegetation Index), cloud, and population data were also converted by interpolation or aggregation methods to match 1-km grid cells.

**Model development and validation:** A gap-filling approach was utilized to produce full-coverage PM_2.5_ concentrations. In brief, two individual models, AOD model (included AOD as a predictor on days and at grid cells with available AOD) and non-AOD model (on all days and at all grid cells) were constructed. Then, AOD model was used to predict PM_2.5_ concentrations when AOD was available, and non-AOD model was used when AOD was unavailable. Finally, PM_2.5_ predictions from these two models were combined to generate full-coverage PM_2.5_ predictions at a 1-km resolution and daily level. For cross-validation, the dataset was randomly divided into ten parts, nine of which were used for training a random forest model and one for making predictions as a testing dataset. The process was repeated ten times, and measurements and corresponding predictions were compared. The overall ten-fold cross-validated R^2^ (root-mean-square error, RMSE) between the full-coverage daily predictions and ground measurements was 0.86 (14.46 μg/m^3^), indicating a high degree of accuracy.

**Table S1.** Average duration and standard deviations for each exposure window

| Period | Start | Stop | Fujian | Hebei | Liaoning | Shanghai | Shanxi | Yunnan |
| --- | --- | --- | --- | --- | --- | --- | --- | --- |
| 1 | 3 months before oocyte retrieval | Gonadotropin stimulation | 77 (2) | 79 (2) | 73 (10) | 78 (3) | 78 (2) | 77 (2) |
| 2 | Gonadotropin stimulation | Oocyte retrieval | 13 (2) | 11 (2) | 17 (10) | 12 (3) | 12 (2) | 13 (2) |
| 3 | 3 months before oocyte retrieval | Oocyte retrieval | 90 (0) | 90 (0) | 90 (0) | 90 (0) | 90 (0) | 90 (0) |
| 4 | Embryo transfer | Serum β-hCG testing | 14 (0) | 14 (1) | 13 (2) | 14 (1) | 10 (2) | 15 (2) |
| 5 | Serum β-hCG testing | Ultrasound testing | 14 (0) | 14 (1) | 15(2) | 14 (1) | 18 (2) | 13 (2) |
| 6 | Embryo transfer | Ultrasound testing | 28 (0) | 28 (0) | 28 (0) | 28 (0) | 28 (0) | 28 (0) |

**Note:** unit: days.

**Table S2.** Demographic and clinical characteristics of the study population in Fujian

| **Variables** | **Missing** | **Baseline** | **Live birth** | **Non-live birth** |
| --- | --- | --- | --- | --- |
|  |  | **(N = 7576)** | **(N = 3459)** | **(N = 4117)** |
| Female age (years) | 0 (0.0%) | 31.1 (3.8) | 30.6 (3.6) | 31.5 (3.9) |
| Female BMI (kg/m^2^) | 2 (0.0%) | 21.6 (2.9) | 21.4 (2.8) | 21.7 (2.9) |
| Female education level | 149 (2.0%) |  |  |  |
| ≤ High school diploma |  | 3683 (48.6%) | 1665 (48.1%) | 2018 (49.0%) |
| Junior college diploma |  | 1463 (19.3%) | 665 (19.2%) | 798 (19.4%) |
| Bachelor's degree |  | 2021 (26.7%) | 947 (27.4%) | 1074 (26.1%) |
| ≥ Master's degree |  | 260 (3.4%) | 113 (3.3%) | 147 (3.6%) |
| Male education level | 144 (1.9%) |  |  |  |
| ≤ High school diploma |  | 3718 (49.1%) | 1719 (49.7%) | 1999 (48.6%) |
| Junior college diploma |  | 1360 (18.0%) | 586 (16.9%) | 774 (18.8%) |
| Bachelor's degree |  | 2051 (27.1%) | 947 (27.4%) | 1104 (26.8%) |
| ≥ Master's degree |  | 303 (4.0%) | 142 (4.1%) | 161 (3.9%) |
| Female smoking status | 7 (0.1%) |  |  |  |
| No |  | 7569 (99.9%) | 3456 (99.9%) | 4113 (99.9%) |
| Female Occupation | 0 (0.0%) |  |  |  |
| Unemployed |  | 2038 (26.9%) | 903 (26.1%) | 1135 (27.6%) |
| Undefined^a^ |  | 5538 (73.1%) | 2556 (73.9%) | 2982 (72.4%) |
| Male smoking status | 17 (0.2%) |  |  |  |
| No |  | 6678 (88.1%) | 3006 (86.9%) | 3672 (89.2%) |
| Yes |  | 881 (11.6%) | 445 (12.9%) | 436 (10.6%) |
| Infertility duration (years) | 0 (0.0%) | 3.59 (2.4) | 3.52 (2.4) | 3.66 (2.5) |
| Infertility type | 2 (0.0%) |  |  |  |
| Primary |  | 4001 (52.8%) | 1869 (54.0%) | 2132 (51.8%) |
| Secondary |  | 3573 (47.2%) | 1589 (45.9%) | 1984 (48.2%) |
| Cause of infertility | 0 (0.0%) |  |  |  |
| Male-related |  | 1306 (17.2%) | 568 (16.4%) | 738 (17.9%) |
| Female-related |  | 4524 (59.7%) | 2077 (60.0%) | 2447 (59.4%) |
| Both-related |  | 519 (6.9%) | 215 (6.2%) | 304 (7.4%) |
| Unexplained |  | 1227 (16.2%) | 599 (17.3%) | 628 (15.3%) |
| Gonadotropin dose (IU) | 18 (0.2%) | 2600 (930) | 2570 (906) | 2640 (950) |
| COH protocols | 0 (0.0%) |  |  |  |
| GnRH Antagonist |  | 1743 (23.0%) | 675 (19.5%) | 1068 (25.9%) |
| GnRH agonist^b^ |  | 5619 (74.2%) | 2717 (78.5%) | 2902 (70.5%) |
| Others^c^ |  | 214 (2.8%) | 67 (1.9%) | 147 (3.6%) |
| Fertility methods | 0 (0.0%) |  |  |  |
| IVF |  | 5903 (77.9%) | 2740 (79.2%) | 3163 (76.8%) |
| ICSI |  | 1673 (22.1%) | 719 (20.8%) | 954 (23.2%) |
| No. of retrieved oocytes | 0 (0.0%) | 12.1 (7.1) | 13.3 (7.3) | 11.2 (6.7) |
| No. of high-quality embryos | 0 (0.0%) | 5.53 (4.1) | 6.39 (4.2) | 4.80 (3.8) |
| Type of embryo transferred | 0 (0.0%) |  |  |  |
| Fresh |  | 4794 (63.3%) | 2116 (61.2%) | 2678 (65.0%) |
| Frozen |  | 2782 (36.7%) | 1343 (38.8%) | 1439 (35.0%) |
| No. of days of embryo culture | 0 (0.0%) |  |  |  |
| 3 |  | 5228 (69.0%) | 2203 (63.7%) | 3025 (73.5%) |
| 5 |  | 2006 (26.5%) | 1148 (33.2%) | 858 (20.8%) |
| 6 |  | 342 (4.5%) | 108 (3.1%) | 234 (5.7%) |
| No. of embryo transferred | 0 (0.0%) |  |  |  |
| 1 |  | 3120 (41.2%) | 1383 (40.0%) | 1737 (42.2%) |
| 2 |  | 4456 (58.8%) | 2076 (60.0%) | 2380 (57.8%) |
| Endometrial thickness (mm) | 0 (0.0%) | 11.4 (2.5) | 11.6 (2.8) | 11.2 (2.2) |
| Season of embryo transfer | 0 (0.0%) |  |  |  |
| Spring |  | 1749 (23.1%) | 799 (23.1%) | 950 (23.1%) |
| Summer |  | 2095 (27.7%) | 972 (28.1%) | 1123 (27.3%) |
| Autumn |  | 2229 (29.4%) | 1062 (30.7%) | 1167 (28.3%) |
| Winter |  | 1503 (19.8%) | 626 (18.1%) | 877 (21.3%) |
| COVID-19 pandemic period | 0 (0.0%) |  |  |  |
| No |  | 4649 (61.4%) | 2033 (58.8%) | 2616 (63.5%) |
| Yes |  | 2927 (38.6%) | 1426 (41.2%) | 1501 (36.5%) |

**Abbreviation:** BMI, body mass index; COH, controlled ovarian hyperstimulation; IVF, *in vitro* fertilization; ICSI, intracytoplasmic sperm injection; No., number.

**Note:** Continuous variables are presented with mean and standard deviation (SD), categorical variables are presented with count and percentage (%).

^a^ Undefined include those employed, but absent from detailed occupations.

^b^ GnRH agonist includes long agonist, ultra-long agonist, short agonist, and ultra-short agonist.

^c^ Other COH protocols include progestin-primed ovarian stimulation protocol (PPOS), luteal-phase ovarian stimulation, and mild stimulation protocols. Spring, March-May; Summer, June-August; Autumn, September-November; Winter, December-February. The COVID-19 pandemic period refers to whether any reproductive outcomes occur after January 1, 2020. “Missing” column refers to the missing data of the baseline.

**Table S3.** Demographic and clinical characteristics of the study population in Hebei

| **Variables** | **Missing** | **Baseline** | **Live birth** | **Non-live birth** |
| --- | --- | --- | --- | --- |
|  |  | **(N = 10,893)** | **(N = 4929)** | **(N = 5964)** |
| Female age (years) | 0 (0.0%) | 30.2 (3.9) | 29.7 (3.7) | 30.6 (4.1) |
| Female BMI (kg/m^2^) | 283 (2.6%) | 23.5 (3.6) | 23.5 (3.6) | 23.6 (3.6) |
| Female education level | 66 (0.6%) |  |  |  |
| ≤ High school diploma |  | 6016 (55.2%) | 2701 (54.8%) | 3315 (55.6%) |
| Junior college diploma |  | 2414 (22.2%) | 1091 (22.1%) | 1323 (22.2%) |
| Bachelor's degree |  | 2008 (18.4%) | 925 (18.8%) | 1083 (18.2%) |
| ≥ Master's degree |  | 389 (3.6%) | 182 (3.7%) | 207 (3.5%) |
| Male education level | 117 (1.1%) |  |  |  |
| ≤ High school diploma |  | 6392 (58.7%) | 2854 (57.9%) | 3538 (59.3%) |
| Junior college diploma |  | 2151 (19.7%) | 987 (20.0%) | 1164 (19.5%) |
| Bachelor's degree |  | 1926 (17.7%) | 879 (17.8%) | 1047 (17.6%) |
| ≥ Master's degree |  | 307 (2.8%) | 143 (2.9%) | 164 (2.7%) |
| Female smoking status | 7 (0.1%) |  |  |  |
| No |  | 10,872 (99.8%) | 4917 (99.8%) | 5955 (99.8%) |
| Yes |  | 14 (0.1%) | 10 (0.2%) | 4 (0.1%) |
| Male smoking status | 7 (0.1%) |  |  |  |
| No |  | 9846 (90.4%) | 4465 (90.6%) | 5381 (90.2%) |
| Yes |  | 1040 (9.5%) | 462 (9.4%) | 578 (9.7%) |
| Female Occupation | 0 (0.0%) |  |  |  |
| Office and other indoor workers |  | 4546 (41.7%) | 2088 (42.4%) | 2458 (41.2%) |
| Factory worker |  | 359 (3.3%) | 154 (3.1%) | 205 (3.4%) |
| Outdoor and cooking workers |  | 1063 (9.8%) | 466 (9.5%) | 597 (10.0%) |
| Unemployed |  | 1744 (16.0%) | 792 (16.1%) | 952 (16.0%) |
| Undefined^a^ |  | 3181 (29.2%) | 1429 (29.0%) | 1752 (29.4%) |
| Infertility duration (years) | 103 (0.9%) | 3.68 (2.7) | 3.54 (2.6) | 3.80 (2.8) |
| Infertility type | 14 (0.1%) |  |  |  |
| Primary |  | 6136 (56.3%) | 2854 (57.9%) | 3282 (55.0%) |
| Secondary |  | 4743 (43.5%) | 2067 (41.9%) | 2676 (44.9%) |
| Cause of infertility | 18 (0.2%) |  |  |  |
| Male-related |  | 2597 (23.8%) | 1285 (26.1%) | 1312 (22.0%) |
| Female-related |  | 3434 (31.5%) | 1477 (30.0%) | 1957 (32.8%) |
| Both-related |  | 4359 (40.0%) | 1913 (38.8%) | 2446 (41.0%) |
| Unexplained |  | 485 (4.5%) | 247 (5.0%) | 238 (4.0%) |
| Gonadotropin dose (IU) | 21 (0.2%) | 2500 (1170) | 2450 (907) | 2550 (1350) |
| COH protocols | 15 (0.1%) |  |  |  |
| GnRH Antagonist |  | 2223 (20.4%) | 872 (17.7%) | 1351 (22.7%) |
| GnRH agonist^b^ |  | 8286 (76.1%) | 3936 (79.9%) | 4350 (72.9%) |
| Others^c^ |  | 369 (3.4%) | 121 (2.5%) | 248 (4.2%) |
| Fertility methods | 26 (0.2%) |  |  |  |
| IVF |  | 8153 (74.8%) | 3604 (73.1%) | 4549 (76.3%) |
| ICSI |  | 2714 (24.9%) | 1319 (26.8%) | 1395 (23.4%) |
| No. of retrieved oocytes | 7 (0.1%) | 13.1 (7.6) | 13.9 (7.6) | 12.5 (7.6) |
| No. of high-quality embryos | 11 (0.1%) | 3.89 (2.5) | 4.22 (2.5) | 3.62 (2.4) |
| Type of embryo transferred | 7 (0.1%) |  |  |  |
| Fresh |  | 7394 (67.9%) | 3258 (66.1%) | 4136 (69.3%) |
| Frozen |  | 3492 (32.1%) | 1669 (33.9%) | 1823 (30.6%) |
| No. of days of embryo culture | 11 (0.1%) |  |  |  |
| 2 |  | 18 (0.2%) | 13 (0.3%) | 5 (0.1%) |
| 3 |  | 10,297 (94.5%) | 4660 (94.5%) | 5637 (94.5%) |
| 4 |  | 154 (1.4%) | 53 (1.1%) | 101 (1.7%) |
| 5 |  | 338 (3.1%) | 176 (3.6%) | 162 (2.7%) |
| 6 |  | 71 (0.7%) | 23 (0.5%) | 48 (0.8%) |
| 7 |  | 4 (0.0%) | 0 (0%) | 4 (0.1%) |
| No. of embryo transferred | 58 (0.5%) |  |  |  |
| 1 |  | 1092 (10.0%) | 349 (7.1%) | 743 (12.5%) |
| 2 |  | 9715 (89.2%) | 4555 (92.4%) | 5160 (86.5%) |
| 3 |  | 28 (0.3%) | 9 (0.2%) | 19 (0.3%) |
| Endometrial thickness (mm) | 687 (6.3%) | 10.5 (2.0) | 10.7 (2.0) | 10.4 (2.0) |
| Season of embryo transfer | 0 (0.0%) |  |  |  |
| Spring |  | 3599 (33.0%) | 1616 (32.8%) | 1983 (33.2%) |
| Summer |  | 3089 (28.4%) | 1450 (29.4%) | 1639 (27.5%) |
| Autumn |  | 2508 (23.0%) | 1175 (23.8%) | 1333 (22.4%) |
| Winter |  | 1697 (15.6%) | 688 (14.0%) | 1009 (16.9%) |
| COVID-19 pandemic period | 0 (0.0%) |  |  |  |
| No |  | 8811 (80.9%) | 4038 (81.9%) | 4773 (80.0%) |
| Yes |  | 2082 (19.1%) | 891 (18.1%) | 1191 (20.0%) |

**Abbreviation:** BMI, body mass index; COH, controlled ovarian hyperstimulation; IVF, *in vitro* fertilization; ICSI, intracytoplasmic sperm injection; No., number.

**Note:** Continuous variables are presented with mean and standard deviation (SD), categorical variables are presented with count and percentage (%).

^a^ Undefined includes self-employed individuals, freelancers, and those employed, but absent from detailed occupations.

^b^ GnRH agonist includes long agonist, ultra-long agonist, short agonist, and ultra-short agonist.

^c^ Other COH protocols include progestin-primed ovarian stimulation protocol (PPOS), luteal-phase ovarian stimulation, and mild stimulation protocols. Spring, March-May; Summer, June-August; Autumn, September-November; Winter, December-February. The COVID-19 pandemic period refers to whether any reproductive outcomes occur after January 1, 2020. “Missing” column refers to the missing data of the baseline.

**Table S4.** Demographic and clinical characteristics of the study population in Liaoning

| **Variables** | **Missing** | **Baseline** | **Live birth** | **Non-live birth** |
| --- | --- | --- | --- | --- |
|  |  | **(N = 2547)** | **(N = 1188)** | **(N = 1359)** |
| Female age (years) | 0 (0.0%) | 32.4 (3.5) | 32.0 (3.4) | 32.7 (3.5) |
| Female BMI (kg/m^2^) | 156 (6.1%) | 22.8 (3.5) | 22.8 (3.5) | 22.9 (3.6) |
| Female education level | 0 (0.0%) |  |  |  |
| ≤ High school diploma |  | 549 (21.6%) | 244 (20.5%) | 305 (22.4%) |
| Junior college diploma |  | 519 (20.4%) | 237 (19.9%) | 282 (20.8%) |
| Bachelor's degree |  | 1145 (45.0%) | 553 (46.5%) | 592 (43.6%) |
| ≥ Master's degree |  | 334 (13.1%) | 154 (13.0%) | 180 (13.2%) |
| Male education level | 0 (0.0%) |  |  |  |
| ≤ High school diploma |  | 579 (22.7%) | 255 (21.5%) | 324 (23.8%) |
| Junior college diploma |  | 489 (19.2%) | 241 (20.3%) | 248 (18.2%) |
| Bachelor's degree |  | 1195 (46.9%) | 565 (47.6%) | 630 (46.4%) |
| ≥ Master's degree |  | 284 (11.2%) | 127 (10.7%) | 157 (11.6%) |
| Female smoking status | 0 (0.0%) |  |  |  |
| No |  | 2526 (99.2%) | 1181 (99.4%) | 1345 (99.0%) |
| Yes |  | 21 (0.8%) | 7 (0.6%) | 14 (1.0%) |
| Male smoking status | 1274 (50.0%) |  |  |  |
| No |  | 1082 (42.5%) | 533 (44.9%) | 549 (40.4%) |
| Yes |  | 191 (7.5%) | 90 (7.6%) | 101 (7.4%) |
| Female Occupation | 0 (0.0%) |  |  |  |
| Office and other indoor workers |  | 1460 (57.3%) | 686 (57.7%) | 774 (57.0%) |
| Factory worker |  | 25 (1.0%) | 8 (0.7%) | 17 (1.3%) |
| Outdoor and cooking workers |  | 116 (4.6%) | 52 (4.4%) | 64 (4.7%) |
| Unemployed |  | 151 (5.9%) | 67 (5.6%) | 84 (6.2%) |
| Undefined^a^ |  | 795 (31.2%) | 375 (31.6%) | 420 (30.9%) |
| Infertility duration (years) | 0 (0.0%) | 3.38 (2.4) | 3.28 (2.3) | 3.46 (2.5) |
| Infertility type | 0 (0.0%) |  |  |  |
| Primary |  | 1707 (67.0%) | 802 (67.5%) | 905 (66.6%) |
| Secondary |  | 840 (33.0%) | 386 (32.5%) | 454 (33.4%) |
| Cause of infertility | 0 (0.0%) |  |  |  |
| Male-related |  | 372 (14.6%) | 177 (14.9%) | 195 (14.3%) |
| Female-related |  | 1197 (47.0%) | 548 (46.1%) | 649 (47.8%) |
| Both-related |  | 907 (35.6%) | 433 (36.4%) | 474 (34.9%) |
| Unexplained |  | 71 (2.8%) | 30 (2.5%) | 41 (3.0%) |
| Gonadotropin dose (IU) | 0 (0.0%) | 2430 (856) | 2370 (850) | 2480 (859) |
| COH protocols | 0 (0.0%) |  |  |  |
| GnRH Antagonist |  | 1498 (58.8%) | 707 (59.5%) | 791 (58.2%) |
| GnRH agonist^b^ |  | 927 (36.4%) | 436 (36.7%) | 491 (36.1%) |
| Others^c^ |  | 122 (4.8%) | 45 (3.8%) | 77 (5.7%) |
| Fertility methods | 12 (0.5%) |  |  |  |
| IVF |  | 1214 (47.7%) | 543 (45.7%) | 671 (49.4%) |
| ICSI |  | 975 (38.3%) | 463 (39.0%) | 512 (37.7%) |
| IVF+ICSI |  | 346 (13.6%) | 174 (14.6%) | 172 (12.7%) |
| No. of retrieved oocytes | 0 (0.0%) | 12.6 (7.5) | 13.1 (7.6) | 12.2 (7.4) |
| No. of high-quality embryos | 0 (0.0%) | 2.06 (2.8) | 2.06 (2.9) | 2.06 (2.8) |
| Type of embryo transferred | 0 (0.0%) |  |  |  |
| Fresh |  | 913 (35.8%) | 387 (32.6%) | 526 (38.7%) |
| Frozen |  | 1634 (64.2%) | 801 (67.4%) | 833 (61.3%) |
| No. of days of embryo culture | 3 (0.1%) |  |  |  |
| 2 |  | 11 (0.4%) | 3 (0.3%) | 8 (0.6%) |
| 3 |  | 835 (32.8%) | 349 (29.4%) | 486 (35.8%) |
| 4 |  | 531 (20.8%) | 200 (16.8%) | 331 (24.4%) |
| 5 |  | 621 (24.4%) | 345 (29.0%) | 276 (20.3%) |
| 6 |  | 536 (21.0%) | 283 (23.8%) | 253 (18.6%) |
| 7 |  | 10 (0.4%) | 7 (0.6%) | 3 (0.2%) |
| No. of embryo transferred | 1 (0.0%) |  |  |  |
| 1 |  | 1383 (54.3%) | 674 (56.7%) | 709 (52.2%) |
| 2 |  | 1146 (45.0%) | 510 (42.9%) | 636 (46.8%) |
| 3 |  | 18 (0.7%) | 4 (0.3%) | 14 (1.0%) |
| Endometrial thickness (mm) | 0 (0.0%) | 11.1 (1.8) | 11.2 (1.7) | 11.0 (1.9) |
| Season of embryo transfer | 0 (0.0%) |  |  |  |
| Spring |  | 629 (24.7%) | 290 (24.4%) | 339 (24.9%) |
| Summer |  | 707 (27.8%) | 335 (28.2%) | 372 (27.4%) |
| Autumn |  | 736 (28.9%) | 356 (30.0%) | 380 (28.0%) |
| Winter |  | 475 (18.6%) | 207 (17.4%) | 268 (19.7%) |
| COVID-19 pandemic period | 0 (0.0%) |  |  |  |
| No |  | 1289 (50.6%) | 555 (46.7%) | 734 (54.0%) |
| Yes |  | 1258 (49.4%) | 633 (53.3%) | 625 (46.0%) |

**Abbreviation:** BMI, body mass index; COH, controlled ovarian hyperstimulation; IVF, *in vitro* fertilization; ICSI, intracytoplasmic sperm injection; No., number.

**Note:** Continuous variables are presented with mean and standard deviation (SD), categorical variables are presented with count and percentage (%).

^a^ Undefined includes self-employed individuals, freelancers, and those employed, but absent from detailed occupations.

^b^ GnRH agonist includes long agonist, ultra-long agonist, short agonist, and ultra-short agonist.

^c^ Other COH protocols include progestin-primed ovarian stimulation protocol (PPOS), luteal-phase ovarian stimulation, and mild stimulation protocols. Spring, March-May; Summer, June-August; Autumn, September-November; Winter, December-February. The COVID-19 pandemic period refers to whether any reproductive outcomes occur after January 1, 2020. “Missing” column refers to the missing data of the baseline.

**Table S5.** Demographic and clinical characteristics of the study population in Shanghai

| **Variables** | **Missing** | **Baseline** | **Live birth** | **Non-live birth** |
| --- | --- | --- | --- | --- |
|  |  | **(N = 3974)** | **(N = 1499)** | **(N = 2475)** |
| Female age (years) | 0 (0.0%) | 32.4 (3.5) | 31.9 (3.2) | 32.8 (3.6) |
| Female BMI (kg/m^2^) | 71 (1.8%) | 22.0 (3.5) | 21.9 (3.5) | 22.0 (3.5) |
| Female education level | 7 (0.2%) |  |  |  |
| ≤ High school diploma |  | 395 (9.9%) | 122 (8.1%) | 273 (11.0%) |
| Junior college diploma |  | 915 (23.0%) | 344 (22.9%) | 571 (23.1%) |
| Bachelor's degree |  | 2046 (51.5%) | 797 (53.2%) | 1249 (50.5%) |
| ≥ Master's degree |  | 611 (15.4%) | 235 (15.7%) | 376 (15.2%) |
| Male education level | 724 (18.2%) |  |  |  |
| ≤ High school diploma |  | 385 (9.7%) | 147 (9.8%) | 238 (9.6%) |
| Junior college diploma |  | 759 (19.1%) | 284 (18.9%) | 475 (19.2%) |
| Bachelor's degree |  | 1509 (38.0%) | 584 (39.0%) | 925 (37.4%) |
| ≥ Master's degree |  | 597 (15.0%) | 238 (15.9%) | 359 (14.5%) |
| Female smoking status | 3221 (81.1%) |  |  |  |
| No |  | 751 (18.9%) | 261 (17.4%) | 490 (19.8%) |
| Yes |  | 2 (0.1%) | 1 (0.1%) | 1 (0.0%) |
| Male smoking status | 3972 (99.9%) |  |  |  |
| No |  | 2 (0.1%) | 0 (0%) | 2 (0.1%) |
| Female Occupation | 0 (0.0%) |  |  |  |
| Office and other indoor workers |  | 2969 (74.7%) | 1147 (76.5%) | 1822 (73.6%) |
| Factory worker |  | 18 (0.5%) | 5 (0.3%) | 13 (0.5%) |
| Outdoor and cooking workers |  | 105 (2.6%) | 43 (2.9%) | 62 (2.5%) |
| Unemployed |  | 278 (7.0%) | 101 (6.7%) | 177 (7.2%) |
| Undefined^a^ |  | 604 (15.2%) | 203 (13.5%) | 401 (16.2%) |
| Infertility duration (years) | 5 (0.1%) | 2.95 (2.1) | 2.84 (2.0) | 3.01 (2.2) |
| Infertility type | 3 (0.1%) |  |  |  |
| Primary |  | 2605 (65.6%) | 1021 (68.1%) | 1584 (64.0%) |
| Secondary |  | 1366 (34.4%) | 477 (31.8%) | 889 (35.9%) |
| Cause of infertility | 3 (0.1%) |  |  |  |
| Male-related |  | 909 (22.9%) | 361 (24.1%) | 548 (22.1%) |
| Female-related |  | 2530 (63.7%) | 947 (63.2%) | 1583 (64.0%) |
| Both-related |  | 466 (11.7%) | 170 (11.3%) | 296 (12.0%) |
| Unexplained |  | 66 (1.7%) | 20 (1.3%) | 46 (1.9%) |
| Gonadotropin dose (IU) | 17 (0.4%) | 2030 (939) | 1990 (907) | 2050 (958) |
| COH protocols | 1 (0.0%) |  |  |  |
| GnRH Antagonist |  | 1401 (35.3%) | 477 (31.8%) | 924 (37.3%) |
| GnRH agonist^b^ |  | 2033 (51.2%) | 839 (56.0%) | 1194 (48.2%) |
| Others^c^ |  | 539 (13.6%) | 183 (12.2%) | 356 (14.4%) |
| Fertility methods | 0 (0.0%) |  |  |  |
| IVF |  | 2690 (67.7%) | 1027 (68.5%) | 1663 (67.2%) |
| ICSI |  | 1143 (28.8%) | 425 (28.4%) | 718 (29.0%) |
| IVF+ICSI |  | 141 (3.5%) | 47 (3.1%) | 94 (3.8%) |
| No. of retrieved oocytes | 0 (0.0%) | 10.7 (6.3) | 11.1 (6.1) | 10.4 (6.5) |
| No. of high-quality embryos | 2 (0.1%) | 2.56 (2.5) | 2.94 (2.4) | 2.33 (2.5) |
| Type of embryo transferred | 0 (0.0%) |  |  |  |
| Fresh |  | 2557 (64.3%) | 1006 (67.1%) | 1551 (62.7%) |
| Frozen |  | 1417 (35.7%) | 493 (32.9%) | 924 (37.3%) |
| No. of days of embryo culture | 0 (0.0%) |  |  |  |
| 2 |  | 138 (3.5%) | 46 (3.1%) | 92 (3.7%) |
| 3 |  | 3551 (89.4%) | 1345 (89.7%) | 2206 (89.1%) |
| 4 |  | 39 (1.0%) | 15 (1.0%) | 24 (1.0%) |
| 5 |  | 167 (4.2%) | 73 (4.9%) | 94 (3.8%) |
| 6 |  | 79 (2.0%) | 20 (1.3%) | 59 (2.4%) |
| No. of embryo transferred | 0 (0.0%) |  |  |  |
| 1 |  | 2296 (57.8%) | 709 (47.3%) | 1587 (64.1%) |
| 2 |  | 1678 (42.2%) | 790 (52.7%) | 888 (35.9%) |
| Endometrial thickness (mm) | 4 (0.1%) | 10.9 (2.3) | 11.1 (2.3) | 10.8 (2.4) |
| Season of embryo transfer | 0 (0.0%) |  |  |  |
| Spring |  | 914 (23.0%) | 344 (22.9%) | 570 (23.0%) |
| Summer |  | 1140 (28.7%) | 424 (28.3%) | 716 (28.9%) |
| Autumn |  | 1167 (29.4%) | 428 (28.6%) | 739 (29.9%) |
| Winter |  | 753 (18.9%) | 303 (20.2%) | 450 (18.2%) |
| COVID-19 pandemic period | 0 (0.0%) |  |  |  |
| No |  | 2764 (69.6%) | 1093 (72.9%) | 1671 (67.5%) |
| Yes |  | 1210 (30.4%) | 406 (27.1%) | 804 (32.5%) |

**Abbreviation:** BMI, body mass index; COH, controlled ovarian hyperstimulation; IVF, *in vitro* fertilization; ICSI, intracytoplasmic sperm injection; No., number.

**Note:** Continuous variables are presented with mean and standard deviation (SD), categorical variables are presented with count and percentage (%).

^a^ Undefined includes self-employed individuals, freelancers, and those employed, but absent from detailed occupations.

^b^ GnRH agonist includes long agonist, ultra-long agonist, short agonist, and ultra-short agonist.

^c^ Other COH protocols include progestin-primed ovarian stimulation protocol (PPOS), luteal-phase ovarian stimulation, and mild stimulation protocols. Spring, March-May; Summer, June-August; Autumn, September-November; Winter, December-February. The COVID-19 pandemic period refers to whether any reproductive outcomes occur after January 1, 2020. “Missing” column refers to the missing data of the baseline.

**Table S6.** Demographic and clinical characteristics of the study population in Shanxi

| **Variables** | **Missing** | **Baseline** | **Live birth** | **Non-live birth** |
| --- | --- | --- | --- | --- |
|  |  | **(N = 4998)** | **(N = 1829)** | **(N = 3169)** |
| Female age (years) | 0 (0.0%) | 30.4 (3.5) | 30.1 (3.4) | 30.6 (3.6) |
| Female BMI (kg/m^2^) | 36 (0.7%) | 23.4 (3.9) | 23.3 (4.1) | 23.4 (3.8) |
| Female education level | 0 (0.0%) |  |  |  |
| ≤ High school diploma |  | 2074 (41.5%) | 716 (39.1%) | 1358 (42.9%) |
| Junior college diploma |  | 1264 (25.3%) | 484 (26.5%) | 780 (24.6%) |
| Bachelor's degree |  | 1430 (28.6%) | 533 (29.1%) | 897 (28.3%) |
| ≥ Master's degree |  | 230 (4.6%) | 96 (5.2%) | 134 (4.2%) |
| Male education level | 0 (0.0%) |  |  |  |
| ≤ High school diploma |  | 2230 (44.6%) | 804 (44.0%) | 1426 (45.0%) |
| Junior college diploma |  | 1301 (26.0%) | 460 (25.2%) | 841 (26.5%) |
| Bachelor's degree |  | 1292 (25.9%) | 488 (26.7%) | 804 (25.4%) |
| ≥ Master's degree |  | 175 (3.5%) | 77 (4.2%) | 98 (3.1%) |
| Female smoking status | 0 (0.0%) |  |  |  |
| No |  | 4995 (99.9%) | 1828 (99.9%) | 3167 (99.9%) |
| Yes |  | 3 (0.1%) | 1 (0.1%) | 2 (0.1%) |
| Male smoking status | 0 (0.0%) |  |  |  |
| No |  | 2817 (56.4%) | 1052 (57.5%) | 1765 (55.7%) |
| Yes |  | 2181 (43.6%) | 777 (42.5%) | 1404 (44.3%) |
| Female Occupation | 0 (0.0%) |  |  |  |
| Office and other indoor workers |  | 2339 (46.8%) | 872 (47.7%) | 1467 (46.3%) |
| Factory worker |  | 194 (3.9%) | 64 (3.5%) | 130 (4.1%) |
| Outdoor and cooking workers |  | 170 (3.4%) | 74 (4.0%) | 96 (3.0%) |
| Unemployed |  | 1658 (33.2%) | 569 (31.1%) | 1089 (34.4%) |
| Undefined^a^ |  | 637 (12.7%) | 250 (13.7%) | 387 (12.2%) |
| Infertility duration (years) | 0 (0.0%) | 3.95 (2.6) | 3.74 (2.5) | 4.07 (2.7) |
| Infertility type | 0 (0.0%) |  |  |  |
| Primary |  | 3123 (62.5%) | 1170 (64.0%) | 1953 (61.6%) |
| Secondary |  | 1875 (37.5%) | 659 (36.0%) | 1216 (38.4%) |
| Cause of infertility | 1 (0.0%) |  |  |  |
| Male-related |  | 833 (16.7%) | 324 (17.7%) | 509 (16.1%) |
| Female-related |  | 3644 (72.9%) | 1324 (72.4%) | 2320 (73.2%) |
| Both-related |  | 156 (3.1%) | 54 (3.0%) | 102 (3.2%) |
| Unexplained |  | 364 (7.3%) | 127 (6.9%) | 237 (7.5%) |
| Gonadotropin dose (IU) | 17 (0.3%) | 3030 (1040) | 3020 (1050) | 3030 (1040) |
| COH protocols | 0 (0.0%) |  |  |  |
| GnRH Antagonist |  | 598 (12.0%) | 231 (12.6%) | 367 (11.6%) |
| GnRH agonist^b^ |  | 4302 (86.1%) | 1573 (86.0%) | 2729 (86.1%) |
| Others^c^ |  | 98 (2.0%) | 25 (1.4%) | 73 (2.3%) |
| Fertility methods | 2 (0.0%) |  |  |  |
| IVF |  | 3540 (70.8%) | 1320 (72.2%) | 2220 (70.1%) |
| ICSI |  | 1451 (29.0%) | 506 (27.7%) | 945 (29.8%) |
| IVF+ICSI |  | 5 (0.1%) | 3 (0.2%) | 2 (0.1%) |
| No. of retrieved oocytes | 3 (0.1%) | 16.3 (9.5) | 17.3 (9.8) | 15.7 (9.4) |
| No. of high-quality embryos | 10 (0.2%) | 5.30 (4.0) | 6.33 (4.2) | 4.71 (3.8) |
| Type of embryo transferred | 0 (0.0%) |  |  |  |
| Fresh |  | 577 (11.5%) | 184 (10.1%) | 393 (12.4%) |
| Frozen |  | 4421 (88.5%) | 1645 (89.9%) | 2776 (87.6%) |
| No. of days of embryo culture | 1 (0.0%) |  |  |  |
| 2 |  | 10 (0.2%) | 3 (0.2%) | 7 (0.2%) |
| 3 |  | 3105 (62.1%) | 1026 (56.1%) | 2079 (65.6%) |
| 4 |  | 266 (5.3%) | 69 (3.8%) | 197 (6.2%) |
| 5 |  | 1384 (27.7%) | 635 (34.7%) | 749 (23.6%) |
| 6 |  | 230 (4.6%) | 95 (5.2%) | 135 (4.3%) |
| 7 |  | 2 (0.0%) | 0 (0%) | 2 (0.1%) |
| No. of embryo transferred | 0 (0.0%) |  |  |  |
| 1 |  | 1065 (21.3%) | 271 (14.8%) | 794 (25.1%) |
| 2 |  | 3929 (78.6%) | 1558 (85.2%) | 2371 (74.8%) |
| 3 |  | 4 (0.1%) | 0 (0%) | 4 (0.1%) |
| Endometrial thickness (mm) | 0 (0.0%) | 9.81 (1.6) | 9.88 (1.6) | 9.76 (1.6) |
| Season of embryo transfer | 0 (0.0%) |  |  |  |
| Spring |  | 958 (19.2%) | 347 (19.0%) | 611 (19.3%) |
| Summer |  | 1702 (34.1%) | 600 (32.8%) | 1102 (34.8%) |
| Autumn |  | 1588 (31.8%) | 615 (33.6%) | 973 (30.7%) |
| Winter |  | 750 (15.0%) | 267 (14.6%) | 483 (15.2%) |
| COVID-19 pandemic period | 0 (0.0%) |  |  |  |
| No |  | 3297 (66.0%) | 1175 (64.2%) | 2122 (67.0%) |
| Yes |  | 1701 (34.0%) | 654 (35.8%) | 1047 (33.0%) |

**Abbreviation:** BMI, body mass index; COH, controlled ovarian hyperstimulation; IVF, *in vitro* fertilization; ICSI, intracytoplasmic sperm injection; No., number.

**Note:** Continuous variables are presented with mean and standard deviation (SD), categorical variables are presented with count and percentage (%).

^a^ Undefined includes self-employed individuals, freelancers, and those employed, but absent from detailed occupations.

^b^ GnRH agonist includes long agonist, ultra-long agonist, short agonist, and ultra-short agonist.

^c^ Other COH protocols include progestin-primed ovarian stimulation protocol (PPOS), luteal-phase ovarian stimulation, and mild stimulation protocols. Spring, March-May; Summer, June-August; Autumn, September-November; Winter, December-February. The COVID-19 pandemic period refers to whether any reproductive outcomes occur after January 1, 2020. “Missing” column refers to the missing data of the baseline.

**Table S7.** Demographic and clinical characteristics of the study population in Yunnan

| **Variables** | **Missing** | **Baseline** | **Live birth** | **Non-live birth** |
| --- | --- | --- | --- | --- |
|  |  | **(N = 28,649)** | **(N = 11,637)** | **(N = 17,012)** |
| Female age (years) | 0 (0.0%) | 30.8 (4.1) | 30.3 (3.9) | 31.2 (4.2) |
| Female BMI (kg/m^2^) | 0 (0.0%) | 22.1 (2.8) | 21.9 (2.7) | 22.2 (2.8) |
| Female education level | 0 (0.0%) |  |  |  |
| ≤ High school diploma |  | 17,369 (60.6%) | 6862 (59.0%) | 10,507 (61.8%) |
| Junior college diploma |  | 4429 (15.5%) | 1851 (15.9%) | 2578 (15.2%) |
| Bachelor's degree |  | 6310 (22.0%) | 2696 (23.2%) | 3614 (21.2%) |
| ≥ Master's degree |  | 541 (1.9%) | 228 (2.0%) | 313 (1.8%) |
| Male education level | 0 (0.0%) |  |  |  |
| ≤ High school diploma |  | 16,969 (59.2%) | 6712 (57.7%) | 10257 (60.3%) |
| Junior college diploma |  | 4543 (15.9%) | 1883 (16.2%) | 2660 (15.6%) |
| Bachelor's degree |  | 6611 (23.1%) | 2830 (24.3%) | 3781 (22.2%) |
| ≥ Master's degree |  | 526 (1.8%) | 212 (1.8%) | 314 (1.8%) |
| Female smoking status | 0 (0.0%) |  |  |  |
| No |  | 28,473 (99.4%) | 11,578 (99.5%) | 16,895 (99.3%) |
| Yes |  | 176 (0.6%) | 59 (0.5%) | 117 (0.7%) |
| Male smoking status | 8350 (29.1%) |  |  |  |
| No |  | 14,928 (52.1%) | 6333 (54.4%) | 8595 (50.5%) |
| Yes |  | 5371 (18.7%) | 2312 (19.9%) | 3059 (18.0%) |
| Female Occupation | 0 (0.0%) |  |  |  |
| Office and other indoor workers |  | 8908 (31.1%) | 3715 (31.9%) | 5193 (30.5%) |
| Factory worker |  | 359 (1.3%) | 144 (1.2%) | 215 (1.3%) |
| Outdoor and cooking workers |  | 3489 (12.2%) | 1355 (11.6%) | 2134 (12.5%) |
| Unemployed ^a^ |  | 12,380 (43.2%) | 5001 (43.0%) | 7379 (43.4%) |
| Undefined |  | 3513 (12.3%) | 1422 (12.2%) | 2091 (12.3%) |
| Infertility duration (years) | 175 (0.6%) | 4.13 (2.8) | 3.94 (2.6) | 4.27 (2.9) |
| Infertility type | 0 (0.0%) |  |  |  |
| Primary |  | 13,573 (47.4%) | 5508 (47.3%) | 8065 (47.4%) |
| Secondary |  | 15,076 (52.6%) | 6129 (52.7%) | 8947 (52.6%) |
| Cause of infertility | 0 (0.0%) |  |  |  |
| Male-related |  | 3190 (11.1%) | 1339 (11.5%) | 1851 (10.9%) |
| Female-related |  | 21,104 (73.7%) | 8517 (73.2%) | 12,587 (74.0%) |
| Both-related |  | 3244 (11.3%) | 1342 (11.5%) | 1902 (11.2%) |
| Unexplained |  | 1111 (3.9%) | 439 (3.8%) | 672 (4.0%) |
| Gonadotropin dose (IU) | 0 (0.0%) | 2240 (908) | 2150 (869) | 2300 (928) |
| COH protocols | 0 (0.0%) |  |  |  |
| GnRH Antagonist |  | 4815 (16.8%) | 1659 (14.3%) | 3156 (18.6%) |
| GnRH agonist^b^ |  | 22,877 (79.9%) | 9732 (83.6%) | 13,145 (77.3%) |
| Others^c^ |  | 957 (3.3%) | 246 (2.1%) | 711 (4.2%) |
| Fertility methods | 0 (0.0%) |  |  |  |
| IVF |  | 23,711 (82.8%) | 9680 (83.2%) | 14,031 (82.5%) |
| ICSI |  | 4938 (17.2%) | 1957 (16.8%) | 2981 (17.5%) |
| No. of retrieved oocytes | 0 (0.0%) | 13.7 (8.1) | 14.8 (8.2) | 12.9 (7.9) |
| No. of high-quality embryos | 0 (0.0%) | 5.87 (4.5) | 6.74 (4.7) | 5.28 (4.3) |
| Type of embryo transferred | 0 (0.0%) |  |  |  |
| Fresh |  | 18,725 (65.4%) | 7165 (61.6%) | 11,560 (68.0%) |
| Frozen |  | 9924 (34.6%) | 4472 (38.4%) | 5452 (32.0%) |
| No. of days of embryo culture | 0 (0.0%) |  |  |  |
| 3 |  | 20,987 (73.3%) | 7925 (68.1%) | 13,062 (76.8%) |
| 5 |  | 7662 (26.7%) | 3712 (31.9%) | 3950 (23.2%) |
| No. of embryo transferred | 0 (0.0%) |  |  |  |
| 1 |  | 4770 (16.6%) | 1080 (9.3%) | 3690 (21.7%) |
| 2 |  | 23,879 (83.4%) | 10,557 (90.7%) | 13,322 (78.3%) |
| Endometrial thickness (mm) | 28 (0.1%) | 8.83 (1.9) | 8.84 (1.8) | 8.82 (1.9) |
| Season of embryo transfer | 0 (0.0%) |  |  |  |
| Spring |  | 6854 (23.9%) | 2764 (23.8%) | 4090 (24.0%) |
| Summer |  | 8811 (30.8%) | 3532 (30.4%) | 5279 (31.0%) |
| Autumn |  | 7491 (26.1%) | 3190 (27.4%) | 4301 (25.3%) |
| Winter |  | 5493 (19.2%) | 2151 (18.5%) | 3342 (19.6%) |
| COVID-19 pandemic period | 0 (0.0%) |  |  |  |
| No |  | 19,585 (68.4%) | 7473 (64.2%) | 12,112 (71.2%) |
| Yes |  | 9064 (31.6%) | 4164 (35.8%) | 4900 (28.8%) |

**Abbreviation:** BMI, body mass index; COH, controlled ovarian hyperstimulation; IVF, *in vitro* fertilization; ICSI, intracytoplasmic sperm injection; No., number.

**Note:** Continuous variables are presented with mean and standard deviation (SD), categorical variables are presented with count and percentage (%).

^a^ Undefined includes self-employed individuals, freelancers, and those employed, but absent from detailed occupations.

^b^ GnRH agonist includes long agonist, ultra-long agonist, short agonist, and ultra-short agonist.

^c^ Other COH protocols include progestin-primed ovarian stimulation protocol (PPOS), luteal-phase ovarian stimulation, and mild stimulation protocols. Spring, March-May; Summer, June-August; Autumn, September-November; Winter, December-February. The COVID-19 pandemic period refers to whether any reproductive outcomes occur after January 1, 2020. “Missing” column refers to the missing data of the baseline.

**Table S8.** The distributions of PM_2.5_ during six exposure periods among the participants undergoing *in vitro* fertilization in six study centers

|  | Mean | SD | Min | P_25_ | Median | P_75_ | Max |
| --- | --- | --- | --- | --- | --- | --- | --- |
| Fujian |  |  |  |  |  |  |  |
| Period1 | 26.33 | 9.22 | 7.86 | 20.17 | 24.82 | 30.74 | 150.60 |
| Period2 | 26.07 | 10.70 | 7.93 | 18.71 | 24.44 | 30.77 | 122.65 |
| Period3 | 26.29 | 9.05 | 8.06 | 20.25 | 24.81 | 30.61 | 140.13 |
| Period4 | 26.33 | 10.92 | 7.91 | 18.79 | 24.71 | 31.41 | 156.26 |
| Period5 | 25.88 | 10.06 | 9.25 | 18.65 | 24.37 | 30.88 | 117.68 |
| Period6 | 25.92 | 9.77 | 10.2 | 18.92 | 24.51 | 30.95 | 115.14 |
| Hebei |  |  |  |  |  |  |  |
| Period1 | 63.86 | 25.15 | 11.95 | 43.36 | 61.83 | 77.76 | 198.04 |
| Period2 | 60.58 | 33.11 | 8.73 | 39.05 | 51.00 | 71.78 | 302.46 |
| Period3 | 63.45 | 24.66 | 11.92 | 43.54 | 61.69 | 76.95 | 193.16 |
| Period4 | 59.39 | 32.22 | 10.35 | 38.14 | 50.76 | 70.02 | 272.78 |
| Period5 | 59.47 | 32.82 | 8.38 | 37.94 | 50.27 | 70.50 | 295.06 |
| Period6 | 59.24 | 31.34 | 9.56 | 37.86 | 51.58 | 69.27 | 258.88 |
| Liaoning |  |  |  |  |  |  |  |
| Period1 | 40.16 | 14.51 | 18.50 | 26.85 | 37.90 | 52.16 | 99.52 |
| Period2 | 40.31 | 19.57 | 10.03 | 24.97 | 35.89 | 49.79 | 162.99 |
| Period3 | 40.13 | 13.97 | 18.70 | 27.54 | 37.55 | 51.95 | 102.51 |
| Period4 | 39.33 | 19.36 | 13.13 | 24.16 | 35.38 | 48.07 | 127.70 |
| Period5 | 39.74 | 19.61 | 12.5 | 24.43 | 35.44 | 48.00 | 118.32 |
| Period6 | 39.60 | 18.68 | 12.82 | 24.68 | 36.59 | 48.83 | 111.95 |
| Shanghai |  |  |  |  |  |  |  |
| Period1 | 33.73 | 9.60 | 13.99 | 26.44 | 32.61 | 40.77 | 61.07 |
| Period2 | 33.40 | 12.58 | 8.94 | 23.79 | 31.68 | 41.24 | 83.64 |
| Period3 | 33.69 | 9.32 | 13.87 | 26.76 | 32.90 | 40.41 | 59.27 |
| Period4 | 33.68 | 12.70 | 9.02 | 23.77 | 32.27 | 41.71 | 82.76 |
| Period5 | 34.49 | 13.47 | 10.19 | 23.83 | 32.38 | 43.42 | 84.35 |
| Period6 | 34.44 | 12.22 | 10.89 | 24.11 | 33.26 | 42.66 | 75.70 |
| Shanxi |  |  |  |  |  |  |  |
| Period1 | 46.15 | 18.39 | 10.19 | 32.97 | 41.99 | 55.05 | 182.68 |
| Period2 | 43.33 | 20.69 | 7.00 | 29.81 | 38.22 | 50.07 | 186.97 |
| Period3 | 45.79 | 17.89 | 9.92 | 33.04 | 41.85 | 54.35 | 176.38 |
| Period4 | 45.10 | 22.23 | 7.14 | 30.56 | 39.45 | 53.20 | 280.61 |
| Period5 | 45.56 | 22.39 | 12.15 | 30.95 | 40.25 | 53.3 | 194.51 |
| Period6 | 45.13 | 21.52 | 10.9 | 30.92 | 39.86 | 53.13 | 212.87 |
| Yunnan |  |  |  |  |  |  |  |
| Period1 | 24.77 | 9.01 | 6.33 | 18.25 | 23.47 | 30.22 | 150.92 |
| Period2 | 23.76 | 10.40 | 5.86 | 16.55 | 21.87 | 29.00 | 220.59 |
| Period3 | 24.62 | 8.79 | 6.29 | 18.34 | 23.34 | 29.92 | 142.20 |
| Period4 | 23.44 | 10.28 | 6.02 | 16.35 | 21.39 | 28.52 | 249.36 |
| Period5 | 23.19 | 10.18 | 5.94 | 16.12 | 21.16 | 28.132 | 165.72 |
| Period6 | 23.24 | 9.87 | 6.92 | 16.28 | 21.27 | 28.24 | 158.4 |

**Abbreviation:** SD, standard deviation; Min, minimum; P_25_, 25^th^ percentile; P_75_, 75^th^ percentile; Max, maximum.

**Note:** unit: μg/m^3^. The definition of exposure periods refers to Fig.1.

**Table S9.** Sensitivity analyses for associations between ambient PM_2.5_ and live birth among patients undergoing *in vitro* fertilization

|  | N | Period 1 | Period 2 | Period3 |
| --- | --- | --- | --- | --- |
| **Main results** | 58,637 | 0.967 (0.939, 0.996) | 0.978 (0.958, 0.998) | 0.966 (0.938, 0.995) |
| **Sensitivity Analysis 1** |  |  |  |  |
| With restrictions on local patients | 54,968 | 0.964 (0.929, 0.999) | 0.976 (0.953, 0.999) | 0.962 (0.927, 0.998) |
| **Sensitivity Analysis 2** |  |  |  |  |
| Male smoking status (unadjusted) ^a^ | 41,853 | 0.984 (0.947, 1.022) | 0.985 (0.960, 1.011) | 0.982 (0.945, 1.021) |
| Male smoking status (adjusted) ^a^ | 41,853 | 0.984 (0.947, 1.022) | 0.985 (0.960, 1.011) | 0.983 (0.945, 1.021) |
| Female smoking status (unadjusted) ^b^ | 44,878 | 0.970 (0.955, 0.984) | 0.983 (0.963, 1.004) | 0.969 (0.953, 0.985) |
| Female smoking status (adjusted) ^b^ | 44,878 | 0.970 (0.955, 0.984) | 0.983 (0.963, 1.004) | 0.969 (0.953, 0.985) |
| **Sensitivity Analysis 3** |  |  |  |  |
| Education level (unadjusted) ^c^ | 53,930 | 0.966 (0.939, 0.995) | 0.977 (0.957, 0.998) | 0.965 (0.938, 0.993) |
| Education level (adjusted) ^c^ | 53,930 | 0.964 (0.938, 0.991) | 0.976 (0.957, 0.995) | 0.963 (0.937, 0.989) |
| **Sensitivity Analysis 4** |  |  |  |  |
| Female occupations (unadjusted) ^d^ | 51,054 | 0.973 (0.940, 1.007) | 0.983 (0.966, 1.001) | 0.972 (0.941, 1.004) |
| Female occupations (adjusted) ^d^ | 51,054 | 0.973 (0.941, 1.006) | 0.983 (0.966, 1.000) | 0.972 (0.942, 1.004) |
| **Sensitivity Analysis 5** |  |  |  |  |
| + NO_2_ concentration | 58,637 | 0.955 (0.938, 0.972) | 0.969 (0.952, 0.987) | 0.951 (0.933, 0.968) |
| + O_3_ concentration | 58,637 | 0.969 (0.952, 0.986) | 0.974 (0.955, 0.993) | 0.966 (0.949, 0.983) |
| **Sensitivity Analysis 6** |  |  |  |  |
| + temperature | 58,637 | 0.955 (0.925, 0.987) | 0.966 (0.942, 0.991) | 0.953 (0.922, 0.984) |

**Note:** Results are presented as odds ratios and 95% confidence intervals for live birth per 10 μg/m^3^ increase in the PM_2.5_ concentration. The definition of exposure periods refers to Fig.1. ^a^ Restricted within Fujian, Hebei, Liaoning, Shanxi, and Yunnan. ^b^ Restricted within Hebei, Liaoning, Shanghai, Shanxi, and Yunnan. ^c^ Both males' and females' education levels were incorporated. ^d^ Restricted within Hebei, Liaoning, Shanghai, Shanxi, and Yunnan. Maximum daily 8 h average (MDA8) ozone is used.

**Table S10.** Pooled odds ratios and 95% confidence intervals for live birth, stratified by season of oocyte retrieval

| Period | Spring (N = 16,321) | Summer (N = 17,727) | Autumn (N = 13,866) | Winter (N = 10,723) |
| --- | --- | --- | --- | --- |
| Period1 | 0.999 (0.974, 1.025) | 0.978 (0.909, 1.052) | 0.978 (0.909, 1.052) | 0.969 (0.926, 1.014) |
| Period2 | 0.965 (0.907, 1.028) | 0.954 (0.901, 1.009) | 0.954 (0.901, 1.009) | 0.973 (0.951, 0.996) |
| Period3 | 0.997 (0.968, 1.027) | 0.970 (0.894, 1.053) | 0.970 (0.894, 1.053) | 0.964 (0.927, 1.002) |


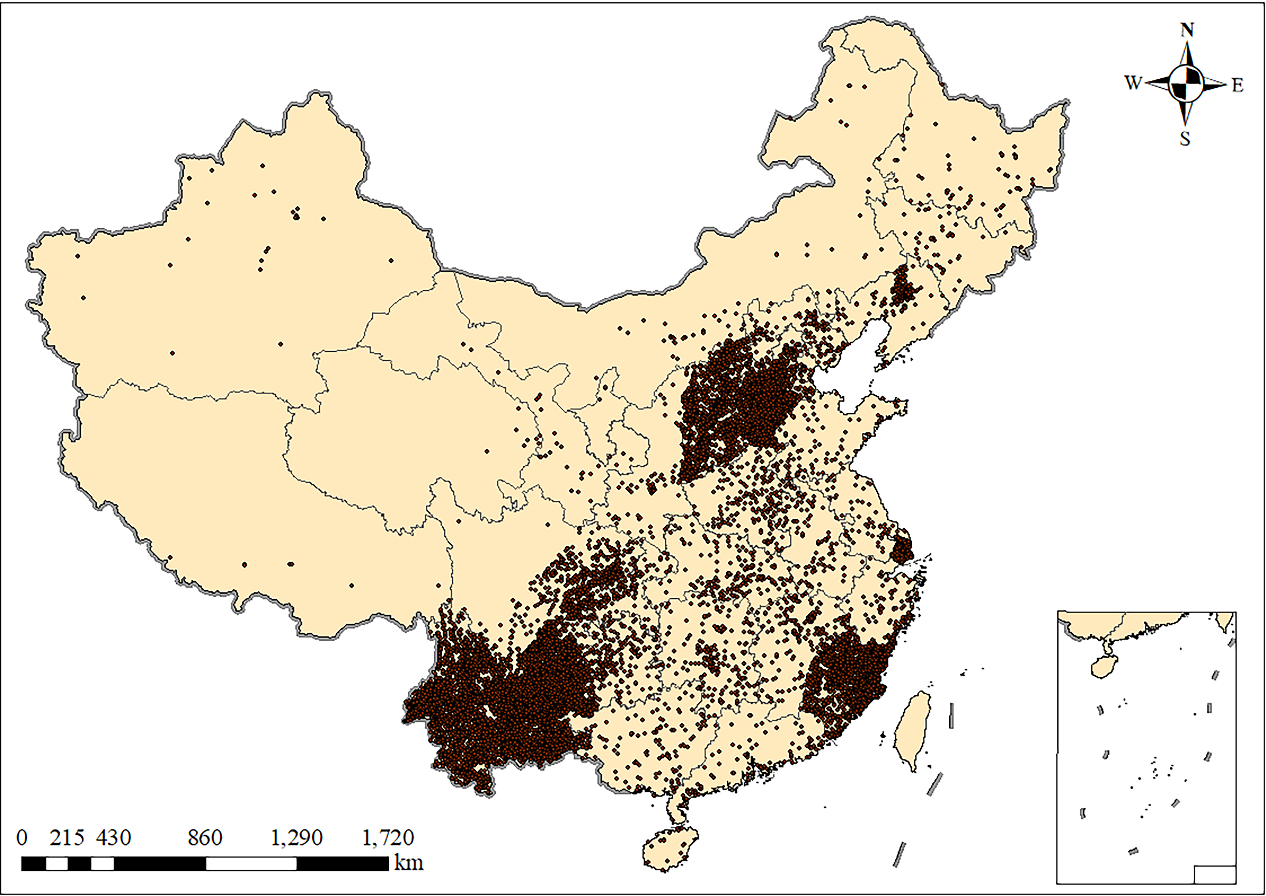


**Fig.S1.** The geographical distribution of study patients

**Note:** Each brown spot refers to the residential address of one patient.

**
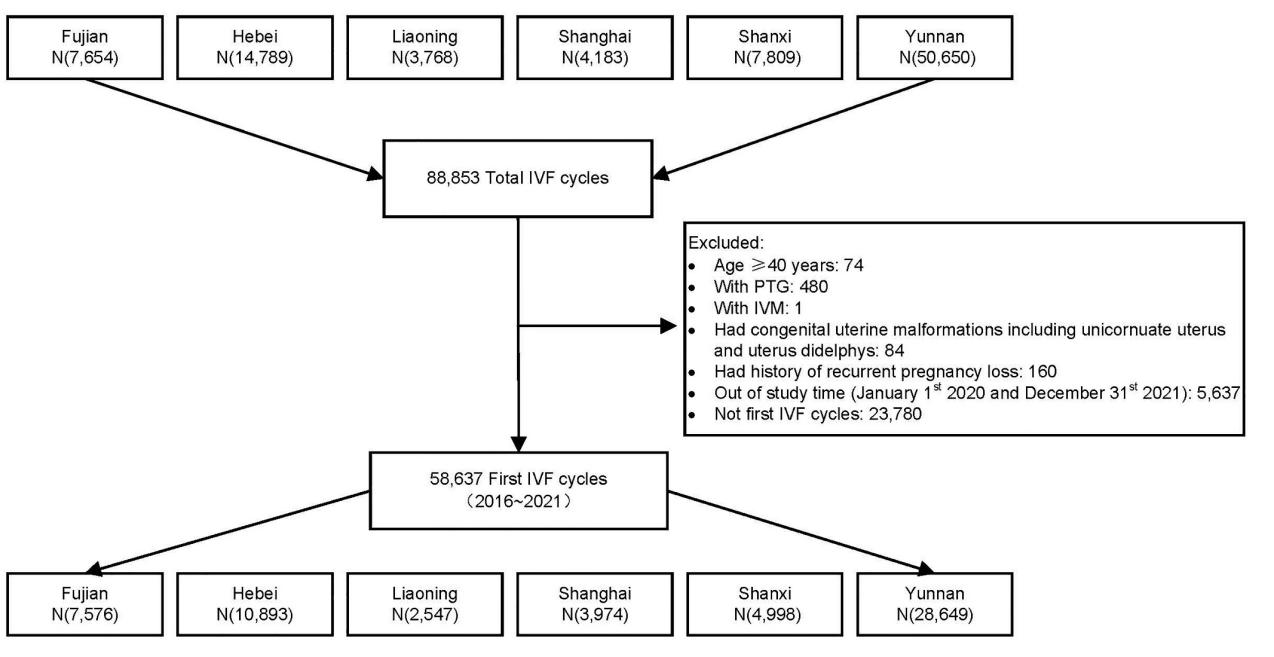
**

**Fig.S2.** Flowchart of *in vitro* cycles included in this study

**Abbreviation:** IVF, *in vitro* fertilization; PGT, preimplantation genetic testing; IVM, *in vitro* maturation.


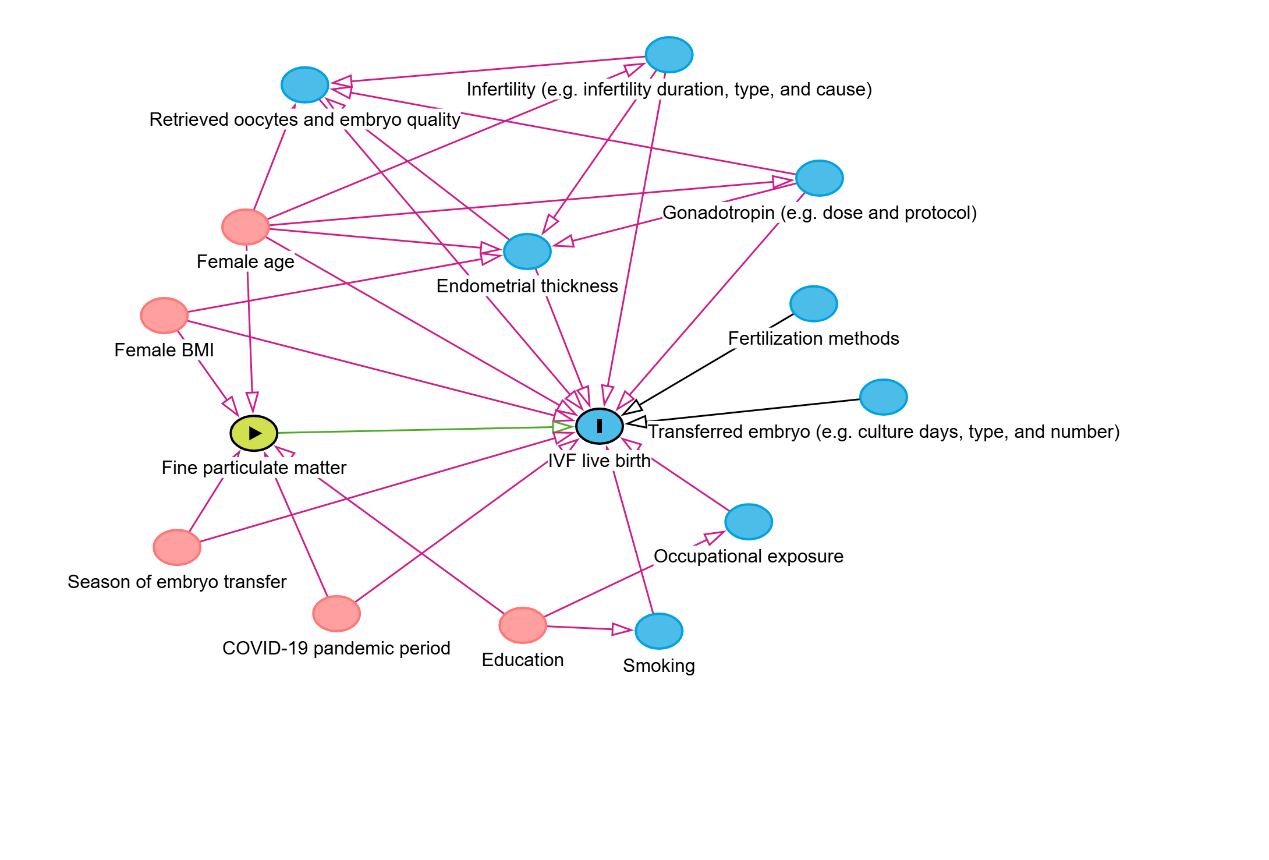


**Fig.S3.** Directed Acyclic Graph for the associations between fine particulate matter pollution and live birth

**Note:** The green circle denotes the exposure, the blue circles denote outcome and ancestor of outcome, and the red circles denote ancestor of exposure and outcome. The green line denotes the causal path between fine particulate pollution and live birth, the red lines denote biasing paths, and the black lines denote the paths between outcome and variable. Minimally sufficient adjustment set: female age, female BMI, education, season of embryo transfer, and COVID-19 pandemic period.

**Abbreviations:** BMI, body mass index, IVF, *in vitro* fertilization.

**Reference**

[1] M. Chen, Y. Wu, X. Huang, W. Li, C. Sun, Z. Meng, et al., Embryo incubation by time-lapse systems versus conventional incubators in Chinese women with diminished ovarian reserve undergoing IVF/ICSI: a study protocol for a randomised controlled trial, BMJ Open. 11 (2020) e038657. <https://doi.org/10.1136/bmjopen-2020-038657>

[2] S. Shi, W. Wang, X. Li, C. Xu, J. Lei, Y. Jiang, et al., Evolution in disparity of PM_2.5_ pollution in China, Eco-Environment & Health. 4 (2023) 257–263. <https://doi.org/10.1016/j.eehl.2023.08.007>

[3] S. Shi, W. Wang, X. Li, Y. Hang, J. Lei, H. Kan, et al., Optimizing modeling windows to better capture the long-term variation of PM_2.5_ concentrations in China during 2005–2019, Science of The Total Environment. 854 (2023) 158624. <https://doi.org/10.1016/j.scitotenv.2022.158624>
